# Evaluation of Two Practical Field Methods for Estimating Operational Overmilking Duration Using Standard Milking-System Sensors

**DOI:** 10.3390/ani16020244

**Published:** 2026-01-13

**Authors:** Alice Uí Chearbhaill, Pablo Silva Boloña, Eoin G. Ryan, Catherine I. McAloon, Martin Browne, John Upton

**Affiliations:** 1 Teagasc, Animal & Grassland Research and Innovation Centre, Moorepark, Fermoy, P61 C997 Cork, Ireland; 2 School of Veterinary Medicine, University College Dublin, Belfield, D04 W6F6 Dublin, Ireland

**Keywords:** overmilking, milking vacuum, milk flow rate, milking quarter

## Abstract

The objective of this study was to quantify the method-to-method variation between two widely used field indicators of the end-of-milking vacuum-exposure period (i.e., operational overmilking duration), and to identify cow- and milking-level factors associated with this variation. Operational overmilking was defined using two approaches: (i) MPC vacuum fluctuation patterns collected via VaDia™ recording devices, and (ii) milk flow curves generated from milking system data, with simulated ACR take-off thresholds ranging from 0.2 to 0.8 kg/min. Seven quarter combinations were analyzed to determine their effect on method-to-method variation. Multivariable modelling was used to investigate the factors which influenced the absolute difference in operational overmilking duration (ADOD) between methods, with larger ADOD indicating greater method-to-method variation. All quarter combinations showed large method-to-method variations. VaDia^TM^-derived estimates indicated longer overmilking durations and higher milk flow at the onset of overmilking compared with the milk flow curve approach. Our findings showed that a combination of the rear quarters was significantly associated with the lowest ADOD, and that a combination of the front quarters was significantly associated with the highest ADOD. All other combinations did not differ from each other, indicating that combinations including one front and one rear quarter performed similarly, and that recording all four quarters did not improve agreement between methods within this dataset. Milk flow factors associated with increased ADOD included longer low flow times, longer high flow times, longer machine-on times, and increased yield. Vacuum values associated with increased ADOD included high short milk tube vacuum during the full milking, and high mouthpiece chamber vacuum levels during both the full milking and overmilking periods. High short milk tube vacuum during overmilking was associated with decreased ADOD. Wider teat diameters, longer teat lengths, and increased parity were associated with increased ADOD. These findings indicated that vacuum-based and flow-based indicators of operational overmilking capture different aspects of the end-milking process and should be clearly specified when measuring or reporting overmilking in research or commercial milking systems.

## Simple Summary

The objective of this study was to evaluate the method-to-method variation between the use of mouthpiece chamber (MPC) vacuum data from VaDia™ recorders and estimates based on simulated automatic cluster remover thresholds using milk flow data to quantify the end-of-milking vacuum-exposure period (i.e., operational overmilking duration) in dairy cows. Overmilking was defined using MPC vacuum fluctuations and milk flow curves at thresholds of 0.2 to 0.8 kg/min. Seven quarter combinations were analyzed, with rear quarters showing the smallest method-to-method differences. These findings demonstrate that vacuum-based and flow-based indicators of operational overmilking capture different aspects of the end-of-milking process and should be clearly specified when measuring or reporting overmilking in research or commercial milking evaluations.

## 1. Introduction

Milk storage in the udder is generally divided into two fractions; the cisternal fraction and the alveolar fraction. Available milk is readily removable from the cisternal fraction; however, it is fixed in the alveolar fraction by capillary forces. The alveolar fraction represents most of the milk stored in the udder, requiring external tactile stimulation and subsequent neuroendocrine reflexes, primarily oxytocin-induced contraction of myoepithelial cells, to expel the milk into the cisternal cavities in a process known as ‘milk ejection’ or ‘milk let-down’ [1]. Once alveolar milk reserves are low or depleted, the flow into the teat cistern diminishes. However, if the milking cluster remains attached and vacuum is still applied to the teat, milk extraction continues; increasingly drawing upon residual cisternal milk at a rate that exceeds resupply. This continued vacuum extraction of depleted reserves results in overmilking, defined as the time when milk flow to the teat cistern is less than the flow out of the teat canal [2]. This physiological definition differs from the operational, system-level definitions commonly used in milking system monitoring, and the present study focuses on the latter.

During overmilking, the absence of milk flow allows for direct transmission of system vacuum to the teat end, increasing mechanical stress and friction. Over time, this can result in impaired blood circulation within the teat tissues [3], increased teat-end thickness [4], and increased occurrence of hyperkeratosis [5], all of which compromise the barrier function of the teat canal. Therefore, field-feasible methods for estimating this end-of-milking vacuum-exposure period are valuable for monitoring milking performance. In this study, this vacuum-exposure period is defined as the operational overmilking duration, which reflects the practical signals available in commercial milking systems rather than the precise physiological onset of alveolar milk depletion.

Traditional flow sensors and automatic cluster removers (ACRs) attempt to address this by detaching the cluster when flow rates drop below a predetermined threshold, commonly a milk flow rate of ≤0.2 kg/min, at which an individual cow is considered sufficiently milked [2]. The literature has shown that increasing ACR thresholds to 0.8 kg/min has no adverse effect on udder health in twice daily milking systems but has significant effects on milking efficiency in pasture-based systems [6,7]. Similarly, other studies indicate that switch-rates as high as 1.2 kg/min have negligible effects on udder health in three-times-daily milking systems, as well as decreased individual animal milking duration [8]. These predetermined thresholds operate solely on external milk outflow (i.e., the rate at which milk is arriving at the milk meter) rather than the internal dynamics of the udder (i.e., the rate at which milk is arriving at the teat cistern from the alveoli). This creates a potential mismatch between perceived and actual milk availability and increases the risk of overmilking, particularly in cows exhibiting bimodality (either through variable milk let-down patterns or incomplete milk ejection) [9] or slow-milking quarters [10]. These thresholds represent operational settings used by milking systems and are not intended to define the physiological onset of overmilking. In this study, they are used solely to generate flow-based estimates for comparison with vacuum-based indicators.

Visualization of real-time pressure differentials across the teat may provide a field-feasible means of identifying when milk inflow becomes insufficient relative to vacuum extraction. This is possible through the documented inverse linear relationship between vacuum and milk flow [11,12]. VaDia^TM^ vacuum recorders (Biocontrol, Rakkestad, Norway) facilitate high-resolution vacuum monitoring at critical points within the milking cluster; specifically, the short milk tube (SMT), short pulse tube (SPT), and mouthpiece chamber (MPC). These locations are useful for understanding the dynamic function of the milking system, as they reflect the complex pressure fluctuations that occur during the milking cycle [13]. Deviations in vacuum profiles can directly influence teat-end health [13], composite milk somatic cell count [14], milk flow rates [15], and overall machine milking performance [16].

Dynamic milking time tests using VaDia^TM^ have been used in the literature to investigate various aspects of milking practices. Wieland et al. [17] used VaDia^TM^ vacuum recorders to successfully estimate bimodal milk ejection using both calculated ‘let-down time’ (defined as the interval between the start of milking, i.e., cluster attachment, and the start of the peak flow period) and visual inspection of traces depicting MPC and teat end vacuum decreasing after the start of milking before markedly increasing again (as defined by Erskine et al. [18]). Vermaak et al. [19] used VaDia^TM^ devices to obtain data for machine-on time and vacuum levels at the SMT (as a proxy for claw vacuum), MPC (front and rear) and SPT (as a proxy for the vacuum in the pulsator chamber), which they used to investigate the effects of different ACR threshold settings (0.5–0.84 kg/min) on cow comfort, overmilking duration, and overmilking claw vacuum.

Despite widespread use of VaDia™ recorders, there is limited guidance on how best to use MPC vacuum data to assess overmilking. Specifically, no studies have examined how overmilking durations derived from MPC vacuum traces vary relative to those estimated from milk flow rate profiles. As a result, it remains unclear how different field-feasible indicators of overmilking relate to one another in practice, or whether they diverge systematically depending on cow- or milking-level factors such as milk-flow dynamics or quarter-level characteristics. This lack of clarity complicates interpretation of vacuum-recording data and limits meaningful comparison of operational overmilking metrics across farms, systems, or studies that rely on different operational definitions. In this study, overmilking was defined using two approaches: (i) MPC vacuum data collected via VaDia™, and (ii) milk flow curves generated from milking system data, with operational overmilking defined as the time after simulated ACR thresholds ranging from 0.2 to 0.8 kg/min until cessation of milk flow. By exploring variation in these approaches across a range of milk-flow cut-off values and quarter configurations, and by identifying factors associated with method-to-method variation, this work advances existing knowledge by clarifying how different definitions of operational overmilking relate to one another in practice. Rather than attempting to define the physiological onset of alveolar milk depletion, this study provides a framework for interpreting vacuum and flow signals as complementary, but not interchangeable, indicators of end-of-milking vacuum exposure in cluster-based milking systems.

The objective of this study was to quantify the method-to-method variation between two field-feasible indicators of operational overmilking duration: (i) MPC-vacuum-based estimates and (ii) milk-flow-based estimates, and to identify cow- and milking-level factors associated with this variation. We also examined how different combinations of recorded quarters (including all-four-quarter configurations) influenced these estimates. This study does not aim to determine the true physiological onset of alveolar milk depletion at the quarter level. Instead, it quantifies how two practical, commercially available indicators differ in estimating the operational end-of-milking vacuum-exposure period in cluster-based milking.

## 2. Materials and Methods

### 2.1. Data Collection

This study was conducted with the approval of the Teagasc Animal Ethics Committee (reference number: TAEC0124/409), and in accordance with the Animal Health and Welfare Act 2013 (updated to 21 May 2025; [20]) and the European Community Directive 86/609/EEC [21].

The study was conducted at the Teagasc Moorepark Research Centre (Fermoy, Co. Cork, Ireland) 46-point Dairymaster (Causeway, Co. Kerry, Ireland) rotary parlour with a low-level milk line over a non-consecutive four-day period in March 2024; i.e., four days across two weeks. The dairy herd operated a seasonal calving system with approximately 330 lactating cows. This study was observational and represents a screening phase of a larger, ethically approved longitudinal experiment for which a priori sample size calculations were performed. All available cows during the recording period were considered for analysis, with the final dataset comprising 102 cows. Cow was considered the experimental unit. All animals were milked twice daily, though data used in this paper was collected during morning milking only. The study herd comprised primarily of Holstein Friesian cows (*n* = 81), with some Jersey (*n* = 19) and HF × Jersey cows (*n* = 2) included.

Twelve VaDia™ (BioControl AS, Rakkestad, Norway) units were used in total in the study. The VaDia^TM^ device captures vacuum curves at a high sampling frequency (200 Hz per channel), enabling measurement of vacuum dynamics within the milking cluster [22]. Two VaDia™ devices were attached to Dairymaster 2.0 kg clusters (one on the front right teatcup and one on the left rear teatcup) fitted with 1022SF liners. Six clusters in total were fitted with VaDia™ devices. These clusters were the same make and model as the clusters used for regular milking. The channels on the VaDia™ devices were set up to record one front and one rear MPC vacuum, and one front and one rear SMT vacuum, each. There were no readings taken for SPT vacuum. The final data recorded for each cluster consisting of four MPC vacuum channels (two front and two rear), and four SMT vacuum channels (two front and two rear).

**Figure 1:**
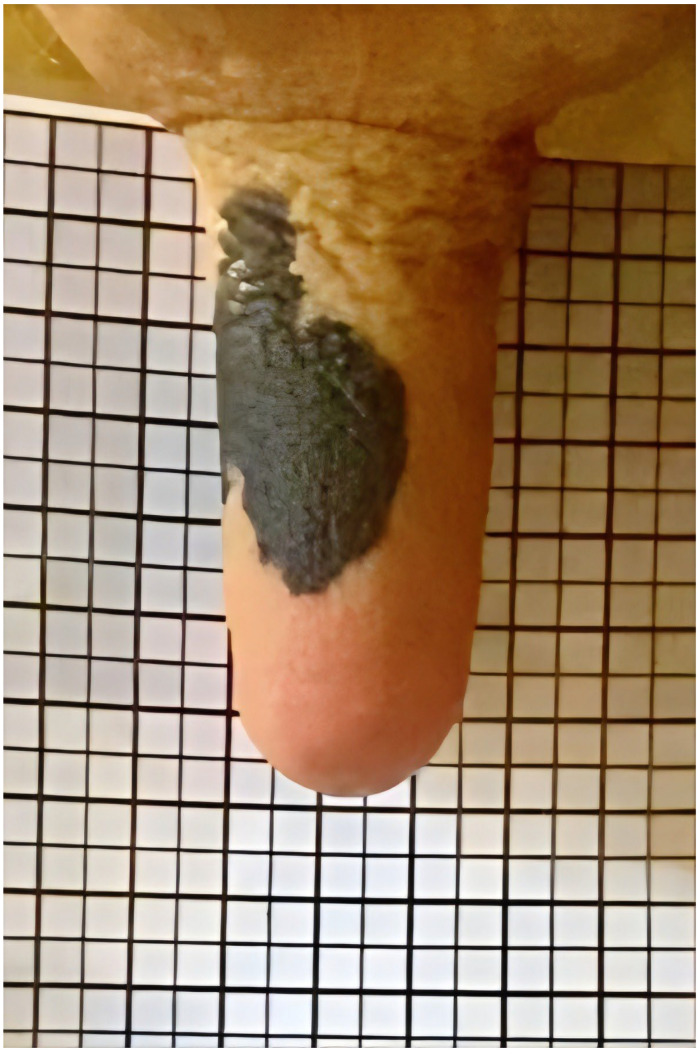
Example of how teat measurements were conducted using 5 mm laminated grid paper.

The clusters were installed in the rotary parlour in place of the normal milking clusters at intervals of seven units. The normal milking clusters were removed and the VaDia™ clusters were attached to the long milk tube, long pulse tube, and ACR cord in such a way as to ensure normal alignment for attachment. Cows entered the rotary in random order for milking as per their normal routine. All cows had their teats measured using a laminated grid (consisting of 5 mm squares) held behind the teat (Figure 1). Each cow’s teat measurements were recorded as smartphone images and later measured digitally for length (from the teat base to the teat end) and diameter (width at the midpoint of the teat barrel) following the method used by Guarín and Ruegg [23]. The ID numbers of cows that entered units with the VaDia™ clusters were manually recorded and visually monitored. Pre-milking teat disinfection was administered to each cow using an automated teat sprayer containing a chlorhexidine-based disinfectant. Teats were then dried with paper towel by one parlour operator before clusters were attached by a second parlour operator. Bluetooth technology monitored the VaDia™ vacuum readings during milking.

Cows with VaDia™ clusters attached were monitored throughout milking to observe the point of cluster removal. The parlour operated a standard ACR take-off setting of 0.2 kg/min, which corresponded with an actual flow rate removal of approximately 0.18 kg/min. Within 10–30 s of VaDia™ cluster removal, each cow was assessed for teat end and teat barrel congestion. Teats were assessed using clean gloves in a clockwise order from the front left to the rear left, with the presence of congestion awarded a nominal score of ‘1’, and absence awarded a nominal score of ‘0’. Observer bias assessment was carried out prior to data collection by ensuring that the observer (AUC) produced similar results to that of a more experienced researcher (JU). Teat end congestion was awarded a score of ‘1’ if the teat end was palpably firm and teat barrel congestion was awarded a score of ‘1’ if there was a palpable ring of swollen tissue at the base of the teat, following the methodology of Mein et al. [24].

All cows underwent post-milking teat disinfection using automated teat sprayers to administer the same chlorhexidine-based disinfectant as was used for pre-milking preparation. Each cluster was also disinfected prior to attachment on the next cow using an automated cluster flush system (ClusterCleanse, Dairymaster, Co. Kerry, Ireland) containing a peracetic acid solution.

Once milking was finished, all VaDia™ clusters were removed, rinsed with water, and dipped in a peracetic acid solution. VaDia™ devices were connected to a laptop and data were acquired using the VaDia™ Suite software (Version 1.16.0.942; BioControl AS, Rakkestad, Norway). In total, 31, 31, 32, and 30 cows were recorded on day one, two, three, and four, respectively (124 cow recordings total).

### 2.2. VaDia™ Biocontrol Measurements

The VaDia™ Suite software was used to generate visualizations and summary data for each recorded cow. Practical markers positioned using the ‘split’ button in the software determined the following criteria: the start of milking (when the SMT vacuum rises above 25 kPa); start of the peak flow period of milking (the midpoint of two 10 s intervals when the average SMT vacuum declines less than 0.15 kPa with increasing milk flow; minimum value of 25 s); start of overmilking (based on an increase in MPC vacuum variation greater or equal to 1.3 times the preceding running average variation); start of cluster take-off (when SMT vacuum is less than 5 kPa below the maximum vacuum at peak flow; working backwards from the end of milking); and end of milking (the first data point with SMT vacuum below 5 kPa after start of the peak flow period) [22] (Section 12.2).

The VaDia™ Suite software provided data for SMT vacuum over the total duration of milking (SMT_TOT_, kPa), the peak flow period of milking (SMT_PFP_, kPa) and the overmilking period (SMT_OM_, kPa). The software provided data for MPC vacuum over the total duration of milking (MPC_TOT_, kPa), the peak flow period of milking (MPC_PFP_, kPa) and the overmilking period (MPC_OM_, kPa). It also provided data for the total milking time (MACHINEON_TIME_, s) and time of overmilking (VD_OM_, s) as defined by the ‘split’ markers. All thresholds, timing criteria, and split-marker definitions used for identification of milking phases and overmilking duration were applied consistently across all cows and recording days in accordance with the VaDia™ Suite User Manual [22].

The quarter-level VaDia™ data were also used to establish which quarter was the slowest to be milked out of the left front (Q1), right front (Q2), right rear (Q3), and left rear (Q4) quarters (SLOWESTQ). This was established based on the quarter with the smallest overmilking time value (SLOWESTQ_TIME_, s).

### 2.3. Milking System Data

Milk yield and milk flow profile data were acquired for each of the four recording days from the Dairymaster rotary parlour software (DairyVue 360; version 1.71.0.4). These data were generated from ICAR-approved milk meters (Dairymaster Weighall Milk Meter, Dairymaster, Co. Kerry, Ireland). The milking parlour data supplied total morning milk yield per cow (YIELD, kg). Milk flow profiles were generated using the cumulative yield (kg) and time (s) outputs, from which the average milk flow rate (AMF, kg/min) was calculated. AMF values were smoothed using a two-point rolling average to reduce signal noise. Operational flow-based variables were derived from the rolling average flow trace. The period of high flow (HIGHFLOW_TIME_, s) was defined as the period when the rolling average flow rate was greater than or equal to 1.5 kg/min, as defined by Upton et al. [25]. The period of low flow (LOWFLOW_TIME_, s) was defined as the period when the rolling average flow rate was greater than 0.2 kg/min but less than 1.5 kg/min. The period of dead time (DEAD_TIME_, s) was defined as the period when the time was less than or equal to 120 s and the rolling average flow rate was less than or equal to 0.2 kg/min. Peak milk flow rate (PMF, kg/min) was calculated by taking the maximum value of each cow’s rolling average flow rates.

Weekly milk recordings also provided data on cow-level somatic cell count (SCC; ×1000 cells/mL).

Flow rate data were used to simulate the effect of different ACR take-off thresholds on milking and operational overmilking time (thresholds used were 0.2, 0.4, 0.6 and 0.8 kg/min). For each threshold, the overmilking duration was calculated where the rolling average flow rate was less than or equal to the specified value after 120 s of machine-on time, to prevent early low flow periods from being misclassified. These durations were labelled as ACR_OM_0.2, ACR_OM_0.4, ACR_OM_0.6, and ACR_OM_0.8, respectively, with time recorded in seconds.

### 2.4. Data Processing

Data for cows that were recorded on more than one day were removed and only their first day of recording was retained to ensure independence of observations. A total of 19 cows were recorded more than once. After removal, 103 cows remained for analysis.

In order to transform the quarter-level VaDia™ data into cow-level data, seven quarter-level combinations were considered. These included the two front quarters (Q12), the two rear quarters (Q34), the left-hand-side quarters (Q14), the right-hand-side quarters (Q23), a combination of the right front and left rear quarter (Q24), a combination of the left front and right rear quarter (Q13), and all four quarters (Q1234).

Teat length and teat diameter data were averaged to obtain cow-level and quarter-combination-level means (TEAT_LENGTH_ and TEAT_DIAMETER_, respectively). All teat end congestion scores were added together to get cow-level and quarter-combination-level teat end congestion scores (TE_CONGESTION_). All teat barrel congestion scores were added together to get cow-level and quarter-combination-level teat barrel congestion scores (TB_CONGESTION_).

All linear models described below assumed independence of observations at the cow level, linearity between predictors and outcome, and approximately normally distributed residuals. Where non-normality was detected, variables were transformed as described below. Data were processed using SAS OnDemand for Academics (https://welcome.oda.sas.com/; accessed on 30 December 2025). Univariate analysis was conducted on all variables to check for normality and for outlier analysis. These procedures were used as model diagnostics to assess distributional assumptions and identify biologically implausible values prior to multivariable modelling. Cow-level SCC was log_10_ transformed due to the non-normal distribution of these data as determined by visual assessment of histograms using the PROC UNIVARIATE (SAS OnDemand) (logSCC). The variables VD_OM_, TE_CONGESTION_, and TB_CONGESTION_ also demonstrated non-normal distribution (Table 1). All reference to VD_OM_ within quarter combinations is considered in terms of median data. Parity was condensed into four groups for ease of analysis (1, 2, 3, ≥4).

To maximize available data, all cows lying outside three standard deviations of the mean for a given variable were assessed to check for biological relevance. One cow was an outlier for multiple variables and was subsequently removed from the study, leaving 102 cows in the final dataset. One cow had her data removed for the SCC variable as she had a cell count of 1,020,000 cells/mL (herd-level mean = 59,676 cells/mL, standard deviation = 118,606 cells/mL) and was later determined to have clinical mastitis. High SCC (>150,000 cells/mL for primiparous cows and >250,000 cells/mL for multiparous cows) has been shown to affect milk flow profiles and milking machine data [26], further justifying her removal as her milking characteristics were highly unlikely to reflect a normal profile for that animal. Two cows had their data removed for LOWFLOW_TIME_ as their values were inconsistent with milking-system behaviour and likely reflected ACR malfunction.

Table 1 shows the results of the univariate analysis for all independent variables.

#### Data Processing; Absolute Difference in Operational Overmilking Duration

The method-to-method absolute difference in operational overmilking duration (ADOD) was calculated for each cow as the absolute difference between the MPC-vacuum–derived operational overmilking duration (VD_OM_; calculated for each quarter combination; VD_OM_Q12, VD_OM_Q34, VD_OM_Q14, VD_OM_Q23, VD_OM_Q24, VD_OM_Q13, VD_OM_Q1234) and the corresponding milk-flow-derived overmilking duration (ACR_OM_; ACR_OM_0.2 to ACR_OM_0.8). These absolute differences formed the ADOD variable (ADOD_VD_OM__ACR_OM_). Average ADOD values for each quarter combination at each simulated ACR threshold are shown in Table 2.

To compare quarter-combination effects, ADOD served as the dependent variable in a mixed-model analysis (PROC MIXED, SAS OnDemand), with quarter combination as the fixed effect. Separate models were fitted for each simulated ACR threshold. Differences between least-squares means were evaluated using model-based pairwise comparisons of estimated marginal means within PROC MIXED (SAS OnDemand).

**Figure 2:**
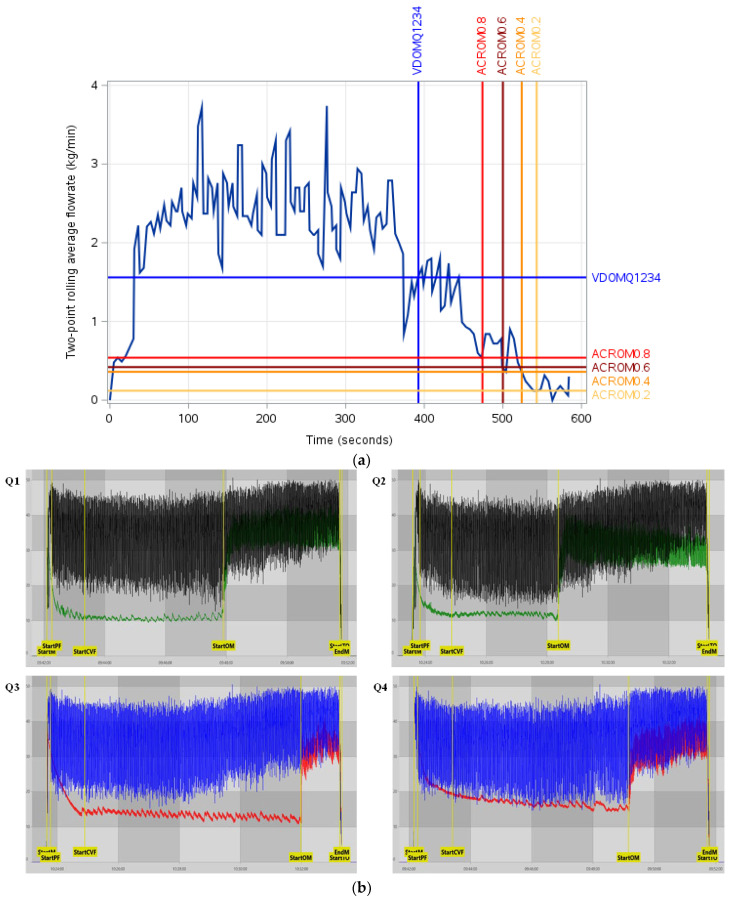
(**a**) Example of milk flow curve with plotted intersections of the time associated with the beginning of overmilking (s) as defined by MPC vacuum fluctuations in the VaDia^TM^ (averaged across all four quarters; Q1234) and four simulated ACR take-off thresholds (0.2–0.8 kg/min) based on milk flow data, with their respective flow rates at each time-point (kg/min). VD_OM_Q1234 = overmilking time as defined by the VaDia^TM^ device for all four quarters, ACR_OM_0.2 = overmilking time associated with an ACR take-off threshold of 0.2 kg/min, ACR_OM_0.4 = overmilking time associated with an ACR take-off threshold of 0.4 kg/min, ACR_OM_0.6 = overmilking time associated with an ACR take-off threshold of 0.6 kg/min, ACR_OM_0.8 = overmilking time associated with an ACR take-off threshold of 0.8 kg/min. (**b**) Quarter-level VaDia^TM^ traces associated with Figure 2a (Q1 = left front, Q2 = right front, Q3 = right rear, Q4 = left rear). StartM = start of milking, StartPF = start of peak flow period, StartCVF = automatic marker set 60 s after ‘StartPF’, StartOM = start of the overmilking period as defined by MPC vacuum, StartTO = start of cluster take-off, EndM = end of milking. For Q1 and Q2 black = SMT Vacuum and Green = MPC Vacuum. For Q3 and Q4 Blue = SMT Vacuum and Red = MPC Vacuum.

For illustration, VD_OM_ and ACR_OM_ were plotted on individual cows’ milk flow curves (flow rate vs. time) to show their positional differences relative to the decline phase of the flow curve. For each threshold, the operational flow-based start point was identified as the moment when the rolling average flow rate fell to or below the simulated threshold after the initial 120 s period. An example of these curves can be observed in Figure 2a, with associated VaDia^TM^ quarter-level traces observable in Figure 2b. Table 3 shows the VD_OM_ and corresponding AMF from the milk flow profiles for different quarter combinations.

### 2.5. Data Analysis

All analyses were conducted in SAS OnDemand for Academics (SAS Institute Inc., Cary, NC, USA). Continuous predictor variables were screened for pairwise collinearity using Pearson correlation coefficients (PROC CORR) [27]. Chi-square tests using PROC FREQ (SAS OnDemand) were conducted between ordinal categorical variables and nominal categorical variables, using Cramer’s V [28]. To investigate the relationships between nominal categorical variables and continuous variables, ANOVAs were conducted when there were ≥3 levels. A non-detectable association (*p* > 0.05) indicated no evidence of a meaningful difference in the dataset. In cases where significance was detected, low R2 values (e.g., <0.2) indicated weak practical relationships. Variables with a high correlation (≥0.8) were not included in the same model.

Seven different mixed models were developed to investigate the association between predictor variables and the ADOD at each of the simulated cut-off time points (28 models total). The model building was carried out in a two-step process using the PROC MIXED procedure (SAS OnDemand). The first step involved univariate analysis (Equation (1)). The second step involved multivariable model building using backwards stepwise elimination. Variables were considered eligible for inclusion in the first iteration of the multivariable model if they had a level of significance of less than or equal to 10% in their univariate analysis; i.e., *p* ≤ 0.1. Variables were removed from each iteration of the multivariable model until all variables remaining in the final model had a level of significance of less than 5%; i.e., *p* < 0.05. This approach ensured parsimonious models while retaining variables with meaningful associations with the outcome.

### 2.6. Model Building—Step 1: Univariate Analysis

The general base model structure is outlined in Equation (1):

ADOD_i_ = β_0_ + β_1_X_i_ + ε; (1)

where ADOD_i_ represents the absolute difference in operational overmilking duration for cow i, and X_i_ represents each predictor variable listed in Table 1. No random or repeated effects were included, as each cow contributed a single observational record to the analysis following removal of repeated recordings.

Independent variables from both the milking system and VaDia™ data were individually introduced into the base model to establish their univariate association with the ADOD. Variables *p* ≤ 0.1 remained for further multivariable analysis.

### 2.7. Model Building—Step 2: Building Initial Models

An initial multivariable linear mixed model (PROC MIXED, SAS OnDemand) for each ADOD quarter and cut-off combination comprised all eligible variables identified from step one.

In cases where predictors exhibited high collinearity (|r| ≥ 0.8), alternative model sets were generated to avoid including collinear variables together. Variance inflation factors (VIF) were assessed using PROC REG (SAS OnDemand) to screen for collinearity, and variables with VIF ≥ 5 were excluded from the multivariable models.

Following collinearity screening, models were refined using iterative removal of variables with the highest *p*-values, following a parsimonious modelling approach commonly used in exploratory analyses. Variables were retained in the final models when *p* < 0.05.

The full list of variables included in the initial models can be found in the Supplementary Materials S1–S7.

## 3. Results

### 3.1. Univariate Analysis of VaDia^TM^ and Milking System Data

Exploratory summaries of VaDia™ and milking system variables are presented in Table 1. The study population comprised mid-parity cows with low median SCC, reflecting generally good udder health status. Considerable between-cow variability was observed in milking duration, milk-flow characteristics, and vacuum exposure, particularly for overmilking-related variables. Notably, vacuum levels increased during the overmilking phase relative to the main milking period, and operational overmilking durations derived from flow-based thresholds increased progressively as ACR cut-off values increased.

### 3.2. VaDia^TM^ Times and Flowrates at VD_OM_

The results of the VD_OM_ times and corresponding AMF at VD_OM_ for all seven quarter combinations can be observed in Table 3. Across all quarter combinations, MPC-vacuum–derived overmilking times (VD_OM_) consistently occurred at substantially higher milk flow rates than those defined by simulated ACR thresholds, resulting in markedly longer vacuum-defined overmilking durations (Table 1 and Table 3). Rear quarters demonstrated the lowest AMF at VD_OM_ and the shortest VD_OM_ durations, whereas front quarters exhibited both the highest AMF at VD_OM_ and the longest vacuum-defined overmilking durations. Mixed front–rear quarter combinations, including the four-quarter combination, showed similar VD_OM_ and AMF values (Table 3). These results demonstrate that MPC-vacuum-based indicators consistently identify the onset of operational overmilking at milk flow levels substantially higher than those defined by simulated ACR thresholds, leading to systematically longer vacuum-defined overmilking durations. Collectively, these findings indicate that vacuum-based indicators systematically identify the onset of operational overmilking earlier in the decline phase of the milk-flow curve than flow-based ACR thresholds, particularly for front quarters.

### 3.3. ADOD

The results for the ADOD of various quarter combinations minus simulated ACR_OM_ thresholds can be observed in Table 2. A lower value for ADOD indicates that the ACR_OM_ threshold values are, on average, closer to the vacuum-derived overmilking values as dictated by VD_OM_. A combination of two rear quarters (i.e., Q34) was associated with the lowest ADOD across all four ACR thresholds, significantly different to all other quarter combinations particularly for the 0.4–0.8 kg/min thresholds (*p* < 0.05). This pattern reflects the shorter vacuum-defined overmilking durations and lower flow rates at VD_OM_ observed for rear quarters. A combination of the front quarters (i.e., Q12) was significantly associated with the highest ADOD across all four ACR thresholds (*p* < 0.05). Q12 had the highest average VD_OM_ (177.3 s) and highest AMF at VD_OM_ (2.3 kg/min). In-keeping with the VaDia™ recommendations of testing one front and one rear MPC vacuum [22] (Section 11.1), there was no significant difference between the ADOD values of any of the combinations involving one front and one rear quarter, including the combination of all four quarters. The ADOD gradually decreased for all quarter combinations as ACR threshold settings increased. Compared to 0.2 kg/min, values at 0.8 kg/min dropped by 36–62% depending on the quarter combination, with the greatest proportional decrease occurring for the rear quarters. Overall, quarter configuration exerted a strong and consistent influence on method-to-method divergence, with front quarters driving the greatest variation between vacuum- and flow-based definitions of operational overmilking.

### 3.4. Multivariable Models

Multivariable modelling identified a stable set of cow-, quarter-, and milking-level factors that consistently influenced the magnitude of divergence between vacuum-based and flow-based overmilking indicators. Across all quarter combinations and simulated ACR thresholds, several consistent patterns emerged in the multivariable models (Table 4, Table 5, Table 6, Table 7, Table 8, Table 9 and Table 10). Longer SLOWESTQ_TIME_ was positively associated with ADOD in every model, indicating that cows with slower-milking quarters showed greater variability between vacuum-based and flow-based estimates of operational overmilking duration. In contrast, higher SMT_OM_ vacuum was consistently associated with lower ADOD across all models.

Flow-related variables showed threshold-dependent effects. Longer HIGHFLOW_TIME_ and LOWFLOW_TIME_ were generally associated with greater ADOD at lower and mid-range thresholds (0.2–0.6 kg/min), but these associations weakened or reversed at 0.8 kg/min. MACHINEON_TIME_ and DEAD_TIME_, when retained, were positively associated with ADOD, though they contributed only in isolated models.

Vacuum variables outside the overmilking period (e.g., SMT_TOT_, SMT_PFP_, MPC_TOT_) and MPC_OM_ were intermittently associated with ADOD depending on threshold and quarter combination, but their direction of effect was consistent: higher vacuum values were associated with greater ADOD. Cow-level traits also contributed, with higher YIELD and longer TEAT_LENGTH_ generally associated with increased ADOD when retained in the models. Wider TEAT_DIAMETER_ was associated with increased ADOD only in the rear-quarter model at 0.2 kg/min (Table 5). Increasing PARITY was associated with increased ADOD only in the all-four-quarter model at 0.2 kg/min (Table 10).

Quarter-combination configuration altered which variables remained in the final models, but the overall pattern was stable: slower-milking quarters, longer flow-period durations, longer machine-on times, higher vacuum exposure, and larger teat dimensions all tended to increase the variation between vacuum-based and flow-based indicators of operational overmilking duration.

Supplementary Tables S1–S4 investigate the factors influencing the first (Qt1) and fourth (Qt4) quartiles of ADOD for the front, rear, left-, and right-hand-side quarter combinations. Cows in Qt4 consistently had the longest durations for LOWFLOW_TIME_, HIGHFLOW_TIME_, VD_OM_, and MACHINEON_TIME_. Cows in Qt4 also had larger YIELD, wider TEAT_DIAMETER_, and lower values for MPC_TOT_ and MPC_OM._ TEAT_LENGTH_ was significantly greater in multiparous than primiparous cows across front, rear, and combined quarters (Supplementary Table S5). TEAT_DIAMETER_ also differed between parities, with differences observed between primiparous and multiparous cows for front teats, and between second- and fourth-or-greater-lactation cows for rear and combined teats (Supplementary Table S6).

## 4. Discussion

This study highlighted clear method-to-method variation in the operational overmilking duration estimated by MPC-vacuum-based and flow-based indicators. These differences reflect the fundamental distinction between the signals used by each method: MPC vacuum responds directly to changes in pressure transmission within the teatcup, whereas flow-based indicators rely on external milk outflow and predefined threshold settings. As such, VD_OM_ and ACR_OM_ capture different operational aspects of the end-of-milking period rather than representing physiologically equivalent events. The magnitude of the variation, visible across all simulated thresholds and quarter configurations, therefore reflects methodological differences rather than deficiencies in either approach. Our findings show that the onset of VD_OM_ consistently precedes the ACR_OM_ thresholds and occurs at a much higher milk flow rate. For example, the average VD_OM_ for all four quarters was 554% higher than the ACR_OM_ at a threshold of 0.2 kg/min, and 77.5% higher than the ACR_OM_ at a threshold of 0.8 kg/min (Table 1 and Table 3), corresponding to an AMF 930% and 157.5% higher, respectively (Table 3). A consolidated summary of model outcomes is provided in Appendix A to support interpretation of the results.

### 4.1. Parity

A parity-related effect on method-to-method variation in operational overmilking duration was observed only under specific conditions, appearing in the all-four-quarter model at the lowest simulated ACR threshold (0.2 kg/min), where higher-parity cows showed greater ADOD. The findings of Tančin et al. [29] showed parity had no influence on peak or average flow rates, the duration of the increase phase nor the decline ratio at either quarter- or cow-level. However, a study by Fernandes et al. [30] found that primiparous cows were subjected to less overmilking time compared to multiparous cows (108.2 s vs. 132.7 s). This supports other findings that primiparous cows can have a significantly shorter decline phase of milk flow than multiparous cows [29,31]. A shorter decline phase likely results in milk flow dropping below the VD_OM_ threshold more quickly, thereby reducing the duration of overmilking and ultimately lowering the ADOD. Increasing parity has been associated with increased teat length and diameter [32], which are known parameters to affect MPC vacuum [12,33] and, therefore, ADOD. A smaller diameter of teat is associated with a greater MPC vacuum [33], which may increase the likelihood of an unclear point for VD_OM_ based on the VaDia^TM^ trace, which in turn contributes to the lower ADOD observed for first lactation animals. This theory is explained in more detail in the ‘Quarter position’ section.

Although teat length and teat diameter differed between parity groups (Supplementary Tables S5 and S6), parity accounted for only a small proportion of the total variation in teat dimensions (R^2^ = 0.08 for teat length; R^2^ = 0.22 for teat diameter), reflecting substantial within-parity variability. As a result, teat dimensions and parity were retained as separate covariates in the multivariable analyses, allowing their associations with method-to-method variation to be evaluated independently.

### 4.2. Quarter Position

Tančin et al. [29] reported that rear quarters had significantly higher milk yield, higher peak and average flow rates, and longer milking times. Front quarters had a shorter duration of increase and decline phases than the rear quarters, but had an overmilking phase that was almost double the duration [29]. The duration of the decline phase is most important as it governs the value for MPC_OM_. This extended overmilking duration in front quarters, observed to be 58.4% higher than rears in our study (Table 3) may explain why the combination of rear quarters had the lowest ADOD (108.5; Table 2). This reduced ADOD could relate to the fact that rear quarters generally have higher mean milk yield than fronts, with Penry et al. [34] demonstrating a difference of approximately 25%. Quarter-yield differences reported in previous studies may contribute to these patterns, but in our dataset all combinations containing one front and one rear quarter showed statistically similar ADOD values. In other words, no mixed front-rear combination outperformed the others, and recording all four quarters did not provide any additional agreement between VD_OM_ and ACR_OM_ within this dataset.

**Figure 3:**
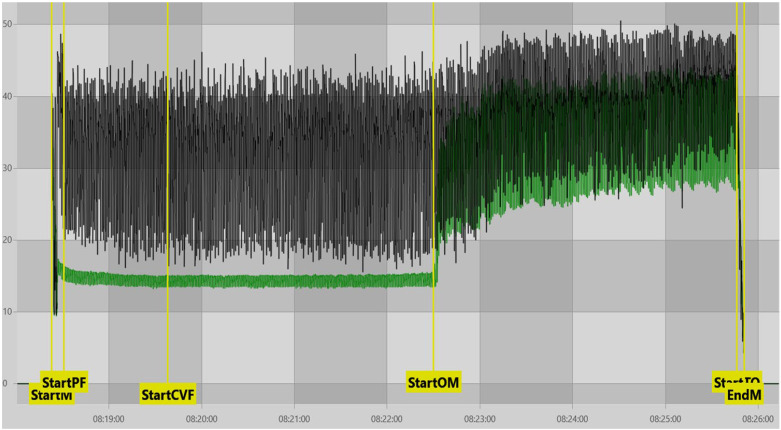
Example of a VaDia^TM^ recording exhibiting clear MPC vacuum phase contrast (green trace = MPC vacuum, black trace = SMT vacuum). StartM = start of milking, StartPF = start of peak flow period, StartCVF = automatic marker set 60 s after ‘StartPF’, StartOM = start of the overmilking period as defined by MPC vacuum, StartTO = start of cluster take-off, EndM = end of milking.

**Figure 4:**
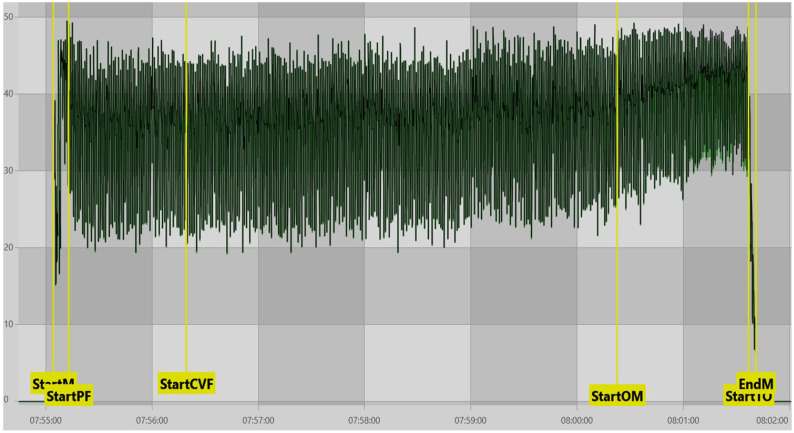
Example of a VaDia^TM^ recording exhibiting poor MPC vacuum phase contrast (green trace = MPC vacuum, black trace = SMT vacuum). StartM = start of milking, StartPF = start of peak flow period, StartCVF = automatic marker set 60 s after ‘StartPF’, StartOM = start of the overmilking period as defined by MPC vacuum, StartTO = start of cluster take-off, EndM = end of milking.

Our findings indicated that front quarters being SLOWESTQ were generally associated with a lower ADOD than the rear quarters being SLOWESTQ. Front quarters generally milk out before the rears due to lower milk yield, resulting in a greater degree of overmilking [35]; findings which were supported in our study (Supplementary Tables S1 and S2). This should, however, lead to an increased ADOD; the opposite of what is observed here. Though the findings of Penry et al. [34] do explain that the front quarters account for the highest or second highest quarter for milk contribution at some point during a lactation in at least 24% of lactations, we do not believe this to be the explanation for our findings. What we propose as more likely is that variation in the interpretability of MPC vacuum traces influenced VD_OM_ determination. Specifically, when MPC vacuum traces exhibited clear phase contrast (more commonly observed during periods of high milk flow or in rear quarters) the onset of operational overmilking (VD_OM_) was identified earlier by the software, resulting in larger ADOD values for those milkings (Figure 3). In contrast, when MPC vacuum remained elevated throughout milking, the visual contrast between peak-flow and overmilking phases was reduced, obscuring the identification of the VD_OM_ timepoint and leading to later detection and lower ADOD values (Figure 4). This reduced phase contrast was more frequently observed under conditions such as bimodal milking, poor liner fit, or low milk yield.

In cases where reduced phase contrast limited automated identification, manual adjustment of the VD_OM_ marker was required. Such adjustments were informed not only by MPC vacuum traces, but also by concurrent evaluation of SMT vacuum behaviour and additional contextual cues, including known milking characteristics of individual cows, consistent with approaches described by Moore-Foster et al. [36]. Supplementary Tables S1–S4 support this interpretation, showing that cows in the lowest ADOD quartile (Qt1) consistently exhibited a higher prevalence of reduced phase-contrast MPC traces, particularly when front quarters were the slowest-milking.

### 4.3. Milking System Values

Higher LOWFLOW_TIME_ was consistently associated with increased ADOD in our study. These extended low flow periods likely represent the tail end of the decline phase, where milk flow drops gradually and eventually falls below 0.2 kg/min. Tančin et al. [29] described how overmilking should be minimized through termination of milking within the decline or low flow stage of the milk flow curve, before the overmilking phase begins. This principle underpins several studies investigating earlier ACR take-off thresholds, aiming to reduce overmilking by setting higher flow cut-off points [6,7,8]. Quartile comparisons also showed that cows with the longest low flow and overmilking periods tended to have the highest ADOD values (Supplementary Tables S1–S4).

Longer HIGHFLOW_TIME_ was also associated with increased ADOD. A study by Upton et al. [25] demonstrated that high flow-rate cows had a smaller reduction in milk yield and milking time compared to low flow-rate cows when they were milked on an ACR take-off setting of 0.8 kg/min compared to 0.2 kg/min. This association with shorter milking time [25,37], could make the risk of overmilking less likely unless quarters differed significantly in the rate at which they milk out [9]. However, as observed in Supplementary Tables S1–S4, the fourth quartile for ADOD not only had the longest duration for high flow time (334.6 s vs. 290.0 s; +15.4%), but also the longest durations for overmilking (168.5 s vs. 70.1 s; +140.5%). This could explain the association with increased ADOD observed. In addition, extended high flow times could indicate a lower MPC vacuum and therefore improved interpretability of the vacuum trace for identification of the timepoint where high flow period comes to an end, triggering the start of VD_OM_ at an earlier point and contributing further to an increased ADOD. In addition, longer high flow time was associated with lower values for MPC_TOT_ and MPC_OM_ (25.2 kPa and 32.0 kPa, respectively; Supplementary Tables S1–S4), conditions that may further enhance phase contrast in MPC vacuum traces and contribute to increased ADOD. Interestingly, the only case where increased high flow time was associated with a decrease in ADOD was for the combined rear quarters at an ACR take-off threshold of 0.8 kg/min. There is no clear explanation for why this was the case.

Larger total machine milking times were associated with increased ADOD. This machine-on time is directly influenced by the duration of overmilking, as overmilking time is a component of the total milking time, as well as dead time. Longer dead time was also associated with increased ADOD. Upton et al. [6] found that reductions in dead time contributed to 12% of the reduction in overall milking time when increasing milk flow-rate switch-points from 0.2 kg/min to 0.8 kg/min. Longer total machine milking times could also be related to increased yield [38], depending on average milk flow rate, which was also associated with increased ADOD. Large yields could delay the time until the ACR take-off milk flow rate is reached and bring forward the initiation of VD_OM_ in instances where there is a long decline phase of milking. Ultimately, both longer total machine time and increased yield reflect the influences of extended low and high flow phases, which helps explain their relationship with elevated ADOD. Fourth quartiles for ADOD were associated with the highest values for MACHINEON_TIME_, YIELD, HIGHFLOW_TIME_, LOWFLOW_TIME_ across all ACR thresholds (Supplementary Tables S1–S4).

### 4.4. VaDia^TM^ Values

Higher SMT_TOT_ vacuums were associated with increased ADOD, whereas higher SMT_OM_ vacuums were associated with decreased ADOD. As reported by Williams et al. [39], the average value for SMT during the overmilking period tends to be higher than during the overall milking period. SMT vacuum tends to display an inverse relationship with milk flow [40], implying that higher SMT vacuum values throughout the entirety of milking could relate to long periods of low flow time and increased machine-on time; both of which were associated with increased ADOD in our study. Large SMT_TOT_ values could also reflect a poor fit between the liner and the teat, specifically when the internal diameter of the liner is too wide, interfering with the teat’s ability to elongate into the liner’s zone of effective compression as milking progresses [41]; contributing further to longer machine-on time. This is of particular importance for shorter teats, as during the rest phase the liner cannot massage them effectively and increases the risk teat end congestion [42]. Higher SMT_OM_ values may reflect the marked drop in milk flow during the overmilking phase, potentially prompting earlier activation of ACR take-off thresholds. This could reduce the duration between VD_OM_ and ACR_OM_ to contribute to lower ADOD values.

Higher MPC_TOT_ and MPC_OM_ vacuums were associated with increased ADOD. The association between MPC_TOT_ and increased ADOD is expected because MPC vacuum contributes directly to the VD_OM_ threshold. The average value for MPC vacuum during the overmilking period is considered greater than for the total milking period, for both front and rear quarters [39]. Therefore, it is not surprising that MPC_OM_ was also associated with increased ADOD based on that explanation. Interestingly, cows in the lowest ADOD quartile had relatively high MPC_TOT_ and MPC_OM_ values (Supplementary Tables S1–S4). This likely reflects the influence of reduced MPC vacuum phase contrast on the identification of VD_OM_, as persistently elevated MPC vacuum during peak flow can obscure the transition into overmilking and necessitate manual adjustment of the VD_OM_ marker.

### 4.5. Teat Parameters

A study by Rønningen [33] showed that the proportion of milkings with a high MPC during the peak flow period was associated with decreasing teat diameter, which is likely explained by poor liner fit. Wider teats may provide a better seal within the mouthpiece chamber, producing clearer MPC traces and likely earlier indication of VD_OM_ due to a more obvious transition to the decline phase of milking. This hypothesis is supported by Supplementary Tables S1–S4, which show that the fourth quartiles for ADOD are associated with wider average teat diameters (30.9 mm vs. 29.7 mm). Borkhus and Rønningen [12] found that teats which penetrated more deeply into the teat cup at the end of peak flow and the beginning of low milk flow were associated with an increase in MPC vacuum. This deeper penetration of teats into the teat cup had an increased occurrence during evening milking, where teats were determined to have a shorter length compared to the start of morning milking [12]. The finding was supported by Rønningen [33], which indicated that the proportion of milkings with a high MPC vacuum during the peak flow period was associated with decreasing teat length. In our study, a plausible explanation for our observed association between longer teat length and increased ADOD is that the longer teats provided a better seal within the liner, provided a clearer signal for the start of the decline phase based on MPC vacuum, and allowed VD_OM_ to be identified at an earlier point. Both findings also relate to the fact that teat length and diameter tend to increase with increasing parity [32], and our study found that increased parity was associated with increased ADOD.

## 5. Conclusions

This study quantified the method-to-method variation between operational overmilking durations defined by MPC vacuum (as measured by the VaDia™ device) and those estimated using flow-based ACR take-off thresholds.

When selecting quarter combinations for VaDia™ recordings, our findings indicate that all tested pairs of one front and one rear quarter, as well as the full set of four quarters, showed no significant differences in ADOD. In contrast, a combination of the rear quarters yielded the significantly lowest ADOD, and a combination of the front quarters yielded the significantly highest ADOD. ADOD was greater for ACR take-off settings of 0.2 kg/min and decreased as take-off settings increased to 0.8 kg/min. Higher low flow times, high flow times, total machine-on times, and increased morning milk yields, were associated with an increased ADOD. High SMT total vacuums, MPC total vacuums, and MPC overmilking vacuums were also associated with increased ADOD. High SMT overmilking vacuums were associated with a decreased ADOD. Increased parity, wider teat diameters and longer teat lengths were associated with increased ADOD.

The two approaches do not measure the same operational signal during the end-of-milking period, and the magnitude of their divergence was associated with milk-flow dynamics and cow- and udder-level characteristics. Importantly, these findings are limited to cow-level operational overmilking measured on a single research farm during four observed morning milking sessions and should not be interpreted as defining the physiological onset of alveolar milk depletion. Establishing physiological overmilking would require quarter-level milk flow and vacuum measurements, which were beyond the scope of this study.

Consistent terminology and explicit reporting of the operational indicator(s) used to define overmilking would support clearer interpretation and comparison of findings across studies.

## Figures and Tables

**Table 1:** Exploratory analysis of VaDia^TM^ (quarter-level) and milking system (cow-level) data.

Variable	Average of Cows	Within Cow Standard Deviation (Mean)	Between Cow Standard Deviation	Within Cow Range (Mean)	Between Cow Range
Cow-level data:					
PARITY	2.60	-	1.51	-	7.00
YIELD (kg)	16.65	-	3.32	-	17.60
SCC (×1000 cells/mL)	28.00 ^1^	-	30.00 ^2^	-	556.00
HIGHFLOW_TIME_ (s)	315.66	-	83.23	-	467.00
LOWFLOW_TIME_ (s)	139.69	-	85.68	-	469.00
DEAD_TIME_ (s)	3.73	-	4.96	-	20.00
ACR_OM_0.2 (s)	22.25	-	9.26	-	48.00
ACR_OM_0.4 (s)	49.83	-	24.87	-	120.00
ACR_OM_0.6 (s)	65.83	-	35.23	-	199.00
ACR_OM_0.8 (s)	82.60	-	46.10	-	265.00
PMF (kg/min)	4.83	-	2.76	-	11.44
AMF (kg/min)	2.01	-	0.55	-	3.52
Quarter-level data:					
MACHINEON_TIME_ (s)	497.71	2.40	121.00	5.28	596.00
VD_OM_ (s)	143.59 ^1^	77.61 ^2^	83.76 ^2^	135.90	414.00
SMT_TOT_ (kPa)	35.90	0.18	1.36	0.39	8.48
SMT_OM_ (kPa)	40.07	1.29	0.96	2.80	5.25
SMT_PFP_ (kPa)	34.26	0.47	2.04	1.03	10.45
MPC_TOT_ (kPa)	27.89	3.25	6.96	7.02	24.35
MPC_OM_ (kPa)	33.85	2.64	3.94	5.84	21.10
MPC_PFP_ (kPa)	24.81	4.22	8.91	9.08	32.55
TEAT_LENGTH_ (mm)	44.80	6.36	8.10	12.32	45.00
TEAT_DIAMETER_ (mm)	30.53	1.29	3.94	2.47	20.00
TB_CONGESTION_	14.71 ^3^	-	-	-	-
TE_CONGESTION_	11.27 ^3^	-	-	-	-

^1^ Median value for SCC and VaDia^TM^ overmilking time. ^2^ Interquartile range for SCC and VaDia^TM^ overmilking time. ^3^ Proportion of individual teats with a positive congestion score (%).

**Table 2:** Method-to-method absolute difference (ADOD) between VaDia^TM^ overmilking times for various quarter combinations (VD_OM_) and overmilking times for simulated ACR threshold values (ACR_OM_) of 0.2 kg/min, 0.4 kg/min, 0.6 kg/min, and 0.8 kg/min.

Quarter Combinations	0.2	0.4	0.6	0.8
Front teats (Q12)	155.04 ^a^	127.48 ^a^	113.54 ^a^	98.71 ^a^
Rear teats (Q34)	108.52 ^c^	62.14 ^c^	49.27 ^c^	41.26 ^c^
Left front and rear (Q14)	125.34 ^b^	97.77 ^b^	84.05 ^b^	71.00 ^b^
Right front and rear (Q23)	119.42 ^bc^	91.85 ^b^	78.25 ^b^	65.11 ^b^
Left font and right rear (Q13)	128.31 ^b^	100.75 ^b^	86.12 ^b^	73.10 ^b^
Right front and left rear (Q24)	116.44 ^bc^	88.87 ^b^	75.41 ^b^	62.43 ^b^
All four quarters (Q1234)	122.38 ^bc^	94.81 ^b^	80.40 ^b^	66.70 ^b^

^a–c^ AD values in the same column with different superscripts differ (*p* < 0.05) for the interaction between ACR threshold value and quarter combination.

**Table 3:** VaDia^TM^ overmilking times (VD_OM_; s) and flow rates (AMF; kg/min) at the point of VD_OM_ across all quarter combinations.

Quarter Combinations	VD_OM_ (s)			AMF at VD_OM_ (kg/min)		
	Mean	Std Dev	Range	Mean	Std Dev	Range
Front teats (Q12)	177.29	77.15	345.50	2.32	1.09	5.83
Rear teats (Q34)	111.96	53.33	257.50	1.82	1.13	5.80
Left front and rear (Q14)	147.58	58.13	291.00	2.11	1.10	5.53
Right front and rear (Q23)	141.66	58.42	270.00	2.00	1.06	5.86
Left font and right rear (Q13)	150.56	63.49	309.00	2.05	1.09	5.97
Right front and left rear (Q24)	138.69	55.36	235.50	2.01	1.06	6.44
All four quarters (Q1234)	143.59	53.96	205.50	2.06	1.06	5.83

**Table 4:** Outputs from the multivariable mixed model showing factors associated with the ADOD between VD_OM_Q12 (s) and ACR_OM_ at 0.2–0.8 (kg/min).

FRONT QUARTERS (Q12)												
	0.2			0.4			0.6			0.8		
Effect	Estimate	Std Err	*p*-Value	Estimate	Std Err	*p*-Value	Estimate	Std Err	*p*-Value	Estimate	Std Err	*p*-Value
Intercept	331.38	116.11	0.0053	556.10	112.84	<0.0001	642.28	112.35	<0.0001	485.36	122.69	0.0001
HIGHFLOW_TIME_	0.13	0.036	0.0007	0.19	0.035	<0.0001	0.21	0.035	<0.0001	-	-	-
LOWFLOW_TIME_	0.20	0.036	<0.0001	0.25	0.036	<0.0001	0.19	0.035	<0.0001	-	-	-
DEAD_TIME_	-	-	-	-	-	-	-	-	-	1.30	0.58	0.027
MPC_OM_	-	-	-	1.67	0.66	0.013	1.63	0.66	0.015	-	-	-
SLOWESTQ_TIME_	0.73	0.046	<0.0001	0.58	0.044	<0.0001	0.52	0.044	<0.0001	0.35	0.050	<0.0001
SMT_TOT_	-	-	-	-	-	-	-	-	-	15.58	3.00	<0.0001
SMT_OM_	−8.93	2.79	0.0019	−16.83	2.90	<0.0001	−19.07	2.88	<0.0001	−26.90	3.37	<0.0001
YIELD	-	-	-	-	-	-	-	-	-	3.61	1.02	0.0006

‘-‘ indicates that the variable was not significant in this final model.

**Table 5:** Outputs from the multivariable mixed model showing factors associated with the ADOD between VD_OM_Q34 (s) and ACR_OM_ at 0.2–0.8 (kg/min).

REAR QUARTERS (Q34)												
	0.2			0.4			0.6			0.8		
Effect	Estimate	Std Err	*p*-Value	Estimate	Std Err	*p*-Value	Estimate	Std Err	*p*-Value	Estimate	Std Err	*p*-Value
Intercept	−227.03	134.64	0.095	689.34	101.52	<0.0001	717.89	93.20	<0.0001	691.78	98.65	<0.0001
HIGHFLOW_TIME_	-	-	-	-	-	-	-	-	-	−0.14	0.032	<0.0001
MACHINEON_TIME_	-	-	-	-	-	-	-	-	-	0.14	0.022	<0.0001
SLOWESTQ_TIME_	0.48	0.059	<0.0001	0.53	0.049	<0.0001	0.43	0.045	<0.0001	0.25	0.048	<0.0001
SMT_TOT_	15.66	2.73	<0.0001	-	-	-	-	-	-	-	-	-
SMT_OM_	−9.91	3.61	0.0002	−22.85	2.82	<0.0001	−23.38	2.59	<0.0001	−17.11	2.38	<0.0001
SMT_PFP_	-	-	-	6.60	1.45	<0.0001	6.19	1.33	<0.0001	-	-	-
TEAT_LENGTH_	-	-	-	0.76	0.31	0.015	0.82	0.28	0.0044	-	-	-
TEAT_DIAMETER_	1.58	0.75	0.039	-	-	-	-	-	-	-	-	-
YIELD	5.32	1.00	<0.0001	-	-	-	-	-	-	-	-	-

‘-‘ indicates that the variable was not significant in this final model.

**Table 6:** Outputs from the multivariable mixed model showing factors associated with the ADOD between VD_OM_Q14 (s) and ACR_OM_ at 0.2–0.8 (kg/min).

LEFT QUARTERS (Q14)												
	0.2			0.4			0.6			0.8		
Effect	Estimate	Std Err	*p*-Value	Estimate	Std Err	*p*-Value	Estimate	Std Err	*p*-Value	Estimate	Std Err	*p*-Value
Intercept	231.80	139.82	0.10	729.88	113.15	<0.0001	778.32	115.60	<0.0001	773.97	106.75	<0.0001
HIGHFLOW_TIME_	-	-	-	0.17	0.040	<0.0001	0.19	0.040	<0.0001	0.23	0.040	<0.0001
LOWFLOW_TIME_	0.14	0.050	0.0073	0.31	0.034	<0.0001	0.22	0.034	<0.0001	0.12	0.032	0.0003
MPC_TOT_	-	-	-	-	-	-	-	-	-	1.16	0.49	0.021
SLOWESTQ_TIME_	0.44	0.054	<0.0001	0.30	0.055	<0.0001	0.26	0.056	<0.0001	0.26	0.052	<0.0001
SMT_TOT_	19.78	4.01	<0.0001	-	-	-	-	-	-	-	-	-
SMT_OM_	−24.19	3.24	<0.0001	−19.98	2.69	<0.0001	−21.33	2.75	<0.0001	−22.76	2.64	<0.0001
TEAT_LENGTH_	-	-	-	0.90	0.35	0.011	0.87	0.35	0.015	1.28	0.38	0.0012
YIELD	5.30	0.99	<0.0001	-	-	-	-	-	-	-	-	-

‘-‘ indicates that the variable was not significant in this final model.

**Table 7:** Outputs from the multivariable mixed model showing factors associated with the ADOD between VD_OM_Q23 (s) and ACR_OM_ at 0.2–0.8 (kg/min).

RIGHT QUARTERS (Q23)												
	0.2			0.4			0.6			0.8		
Effect	Estimate	Std Err	*p*-Value	Estimate	Std Err	*p*-Value	Estimate	Std Err	*p*-Value	Estimate	Std Err	*p*-Value
Intercept	131.37	104.54	0.21	728.13	110.79	<0.0001	814.14	120.30	<0.0001	770.61	122.70	<0.0001
HIGHFLOW_TIME_	-	-	-	0.19	0.038	<0.0001	0.15	0.041	0.018	0.17	0.041	<0.0001
LOWFLOW_TIME_	-	-	-	0.22	0.030	<0.0001	0.18	0.031	<0.0001	0.11	0.032	0.0010
MPC_TOT_	-	-	-	-	-	-	-	-	-	1.21	0.47	0.011
MPC_OM_	-	-	-	2.20	0.72	0.0028	1.84	0.79	0.022	-	-	-
SLOWESTQ												
RIGHT FRONT	−17.71	4.54	0.0002	−15.22	5.21	0.0044	−13.33	5.50	0.018	-	-	-
RIGHT REAR	Reference	.	.	Reference	.	.	Reference	.	.	-	-	-
SLOWESTQ_TIME_	0.67	0.039	<0.0001	0.55	0.049	<0.0001	0.37	0.055	<0.0001	0.25	0.055	<0.0001
SMT_TOT_	18.79	1.91	<0.0001	-	-	-	-	-	-	-	-	-
SMT_OM_	−20.25	2.61	<0.0001	−21.22	2.84	<0.0001	−23.21	3.03	<0.0001	−21.69	2.99	<0.0001
TEAT_LENGTH_	-	-	-	-	-	-	-	-	-	0.76	0.35	0.034
YIELD	4.01	0.77	<0.0001	-	-	-	0.61	0.33	0.070			

‘-‘ indicates that the variable was not significant in this final model.

**Table 8:** Outputs from the multivariable mixed model showing factors associated with the ADOD between VD_OM_Q24 (s) and ACR_OM_ at 0.2–0.8 (kg/min).

RIGHT FRONT AND LEFT REAR QUARTERS (Q24)												
	0.2			0.4			0.6			0.8		
Effect	Estimate	Std Err	*p*-Value	Estimate	Std Err	*p*-Value	Estimate	Std Err	*p*-Value	Estimate	Std Err	*p*-Value
Intercept	124.43	134.60	0.36	858.62	128.21	<0.0001	820.93	126.42	<0.0001	856.82	127.47	<0.0001
HIGHFLOW_TIME_	-	-	-	-	-	-	0.20	0.043	<0.0001	0.083	0.035	0.020
LOWFLOW_TIME_	-	-	-	0.12	0.021	<0.0001	0.18	0.025	<0.0001	-	-	-
MACHINEON_TIME_	-	-	-	-	-	-	-	-	-	0.11	0.023	<0.0001
MPC_TOT_	-	-	-	-	-	-	-	-	-	1.49	0.52	0.0049
MPC_OM_	-	-	-	-	-	-	2.49	0.94	0.0098	-	-	-
SLOWESTQ												
RIGHT FRONT	-	-	-	−25.37	6.19	<0.0001	-	-	-	-	-	-
LEFT REAR	-	-	-	Reference	.	.	-	-	-	-	-	-
SLOWESTQ_TIME_	0.49	0.055	<0.0001	0.43	0.062	<0.0001	0.32	0.055	<0.0001	0.23	0.055	<0.0001
SMT_TOT_	21.05	2.33	<0.0001	-	-	-	-	-	-	-	-	-
SMT_OM_	−22.36	3.34	<0.0001	−20.61	3.15	<0.0001	−24.80	3.28	<0.0001	−24.31	3.17	<0.0001
TEAT_LENGTH_	-	-	-	-	-	-	1.10	0.37	0.0083	0.93	0.39	0.019
YIELD	5.21	0.91	<0.0001	-	-	-	-	-	-	-	-	-

‘-‘ indicates that the variable was not significant in this final model.

**Table 9:** Outputs from the multivariable mixed model showing factors associated with the ADOD between VD_OM_Q13 (s) and ACR_OM_ at 0.2–0.8 (kg/min).

LEFT FRONT AND RIGHT REAR QUARTERS (Q13)												
	0.2			0.4			0.6			0.8		
Effect	Estimate	Std Err	*p*-Value	Estimate	Std Err	*p*-Value	Estimate	Std Err	*p*-Value	Estimate	Std Err	*p*-Value
Intercept	225.98	130.74	0.087	708.92	106.61	<0.0001	808.97	108.92	<0.0001	790.70	99.29	<0.0001
HIGHFLOW_TIME_	-	-	-	0.15	0.038	0.0002	0.17	0.039	<0.0001	0.21	0.037	<0.0001
LOWFLOW_TIME_	0.13	0.048	0.0069	0.26	0.036	<0.0001	0.20	0.036	<0.0001	0.089	0.034	0.012
DEAD_TIME_	-	-	-	1.21	0.59	0.042	-	-	-	1.09	0.55	0.049
SLOWESTQ_TIME_	0.59	0.051	<0.0001	0.44	0.053	<0.0001	0.34	0.054	<0.0001	0.30	0.049	<0.0001
SMT_TOT_	15.78	3.92	0.0001	-	-	-	-	-	-	-	-	-
SMT_OM_	−20.44	3.17	<0.0001	−18.59	2.56	<0.0001	−21.07	2.62	<0.0001	−22.02	2.55	<0.0001
YIELD	4.42	0.95	<0.0001	-	-	-	-	-	-	-	-	-

‘-‘ indicates that the variable was not significant in this final model.

**Table 10:** Outputs from the multivariable mixed model showing factors associated with the ADOD between VD_OM_Q1234 (s) and ACR_OM_ at 0.2–0.8 (kg/min).

ALL QUARTERS (Q1234)												
	0.2			0.4			0.6			0.8		
Effect	Estimate	Std Err	*p*-Value	Estimate	Std Err	*p*-Value	Estimate	Std Err	*p*-Value	Estimate	Std Err	*p*-Value
Intercept	205.55	134.97	0.13	607.46	124.87	<0.0001	751.61	126.10	<0.0001	360.17	111.42	0.0017
HIGHFLOW_TIME_	-	-	-	0.15	0.037	<0.0001	0.17	0.038	<0.0001	-	-	-
LOWFLOW_TIME_	0.15	0.044	0.0006	0.29	0.031	<0.0001	0.22	0.031	<0.0001	−0.090	0.036	0.015
DEAD_TIME_	-	-	-	-	-	-	-	-	-	0.98	0.43	0.026
PARITY		ME:	0.0071	-	-	-	-	-	-	-	-	-
1	−39.12	11.89	0.0014	-	-	-	-	-	-	-	-	-
2	−12.89	7.96	0.11	-	-	-	-	-	-	-	-	-
3	−3.57	7.58	0.64	-	-	-	-	-	-	-	-	-
4+	Reference	.	.	-	-	-	-	-	-	-	-	-
SLOWESTQ	-	-	-		ME:	0.012	-	-	-	-	-	-
LEFT FRONT	-	-	-	−20.60	8.03	0.012	-	-	-	-	-	-
RIGHT FRONT	-	-	-	−24.62	8.12	0.0032	-	-	-	-	-	-
RIGHT REAR	-	-	-	−6.49	6.33	0.31	-	-	-	-	-	-
LEFT REAR	-	-	-	Reference	.	.	-	-	-	-	-	-
SLOWESTQ_TIME_	0.56	0.054	<0.0001	0.38	0.061	<0.0001	0.25	0.060	<0.0001	0.18	0.044	0.0001
SMT_TOT_	20.98	3.68	<0.0001	-	-	-	-	-	-	19.48	2.93	<0.0001
SMT_OM_	−25.66	4.029	<0.0001	−15.54	3.04	<0.0001	−20.12	3.00	<0.0001	−26.97	2.93	<0.0001
TEAT_LENGTH_	-	-	-	-	-	-	0.71	0.33	0.033	-	-	-
YIELD	3.39	1.095	0.0026	-	-	-	-	-	-	5.044	0.72	<0.0001

‘-‘ indicates that the variable was not significant in this final model. ‘ME’ refers to the significance of the main effect of the variable based on the Type III fixed effects.

## Data Availability

The original contributions presented in this study are included in the article. Further inquiries can be directed to the corresponding author.

## Supplementary Materials

The following supporting information can be downloaded at: https://www.mdpi.com/article/10.3390/ani16020244/s1. Material S1: Front quarters (Q12), Material S2: Rear quarters (Q34), Material S3: Left quarters (Q14), Material S4: Right quarters (Q23), Material S5: Front right and rear left quarters (Q24), Material S6: Front left and rear right quarters (Q13), Material S7: All four quarters (Q1234); Table S1: Investigation of the factors influencing the first (Qt1) and fourth (Qt4) quartiles of ADOD for the front quarters (Q12) at simulated ACR take-off thresholds of 0.2–0.8 kg/min, Table S2: Investigation of the factors influencing the first (Qt1) and fourth (Qt4) quartiles of ADOD for the rear quarters (Q34) at simulated ACR take-off thresholds of 0.2–0.8 kg/min, Table S3: Investigation of the factors influencing the first (Qt1) and fourth (Qt4) quartiles of ADOD for the left-hand-side quarters (Q14) at simulated ACR take-off thresholds of 0.2–0.8 kg/min, Table S4: Investigation of the factors influencing the first (Qt1) and fourth (Qt4) quartiles of ADOD for the right-hand-side quarters (Q23) at simulated ACR take-off thresholds of 0.2–0.8 kg/min, Table S5: Teat length measurements (mm) by parity group (1, 2, 3, ≥4) for front quarters (Q12), rear quarters (Q34), and all four quarters (Q1234), Table S6: Teat diameter measurements (mm) by parity group (1, 2, 3, ≥4) for front quarters (Q12), rear quarters (Q34), and all four quarters (Q1234)

## Abbreviations

The following abbreviations are used in this manuscript:

ACR(s) = Automatic cluster remover(s), ACR_OM_0.2 = Overmilking time associated with an ACR take-off threshold of 0.2 kg/min generated from milk flow curve data (s), ACR_OM_0.4 = Overmilking time associated with an ACR take-off threshold of 0.4 kg/min generated from milk flow curve data (s), ACR_OM_0.6 = Overmilking time associated with an ACR take-off threshold of 0.6 kg/min generated from milk flow curve data (s), ACR_OM_0.8 = Overmilking time associated with an ACR take-off threshold of 0.8 kg/min generated from milk flow curve data (s), ACR_OM_ = Simulated ACR take-off time values generated from milk flow curve data (s), ADOD = Method-to-method absolute difference in operational overmilking duration, AMF = Average milk flow rate (kg/min), DEAD_TIME_ = Period of dead time (s), HIGHFLOW_TIME_ = Period of high milk flow (s), logSCC = Logarithmic-10 transformed somatic cell count, LOWFLOW_TIME_ = Period of low milk flow (s), MACHINEON_TIME_ = Total milking time (s), MPC = Mouthpiece chamber, MPC_OM_ = Mouthpiece chamber vacuum during overmilking (kPa), MPC_PFP_ = Mouthpiece chamber vacuum during peak flow period (kPa), MPC_TOT_ = Mouthpiece chamber vacuum during total duration of milking (kPa), PMF = Peak milk flow rate (kg/min), Q1 = Left front quarter, Q2 = Right front quarter, Q3 = Right rear quarter, Q4 = Left rear quarter, Q12 = Front quarters, Q13 = Combination of the left front and right rear quarters, Q14 = Left-hand-side quarters, Q1234 = Combination of all four quarters, Q23 = Right-hand-side quarters, Q24 = Combination of the right front and left rear quarters, Q34 = Rear quarters, SLOWESTQ = Slowest milking quarter, SLOWESTQ_TIME_ = Overmilking time associated with slowest milking quarter (s), SMT = Short milk tube, SMT_OM_ = Short milk tube vacuum during overmilking (kPa), SMT_PFP_ = Short milk tube vacuum during peak flow period (kPa), SMT_TOT_ = Short milk tube vacuum during total duration of milking (kPa), SPT = Short pulse tube, TB_CONGESTION_ = Teat barrel congestion score, TEAT_DIAMETER_ = Measurement of teat diameter (mm), TE_CONGESTION_ = Teat end congestion score, TEAT_LENGTH_ = Measurement of teat length (mm), VD_OM_ = Operational overmilking duration as defined by MPC-vacuum-based split markers (s).

## Appendix A

**Table A1:** Summary of Directions of Association of ADOD Between VDOM Times for Different Quarter Combinations and ACROM Thresholds (0.2–0.8 kg/min).

Variable	Front (Q12)		Rear (Q34)		Left (Q14)		Right (Q23)		RF, LR (Q24)		LF, RR (Q13)		ALL FOUR (Q1234)	
HIGHFLOW_TIME_	**↑**	0.2 0.4 0.6	↓	0.8	↑	0.4 0.6 0.8	↑	0.4 0.6 0.8	↑	0.6 0.8	↑	0.4 0.6 0.8	↑	0.4 0.6
LOWFLOW_TIME_	**↑**	0.2 0.4 0.6			**↑**	0.2 0.4 0.6 0.8	↑	0.4 0.6 0.8	↑	0.4 0.6	**↑**	0.2 0.4 0.6 0.8	**↑** ↓	0.2 0.4 0.6 0.8
DEAD_TIME_	**↑**	0.8									**↑**	0.4 0.8	**↑**	0.8
MACHINEON_TIME_			**↑**	0.8					**↑**	0.8				
MPC_TOT_					**↑**	0.8	**↑**	0.8	**↑**	0.8				
MPC_OM_	**↑**	0.4 0.6					**↑**	0.4 0.6	**↑**	0.6				
SLOWESTQ (Rear → Front)							↓	0.2 0.4 0.6	↓	0.4			↓	0.4
SLOWESTQ_TIME_	**↑**	0.2 0.4 0.6 0.8	**↑**	0.2 0.4 0.6 0.8	**↑**	0.2 0.4 0.6 0.8	**↑**	0.2 0.4 0.6 0.8	**↑**	0.2 0.4 0.6 0.8	**↑**	0.2 0.4 0.6 0.8	**↑**	0.2 0.4 0.6 0.8
SMT_TOT_	**↑**	0.8	**↑**	0.2	**↑**	0.2	**↑**	0.2	**↑**	0.2	**↑**	0.2	**↑**	0.2 0.8
SMT_OM_	↓	0.2 0.4 0.6 0.8	↓	0.2 0.4 0.6 0.8	↓	0.2 0.4 0.6 0.8	↓	0.2 0.4 0.6 0.8	↓	0.2 0.4 0.6 0.8	↓	0.2 0.4 0.6 0.8	↓	0.2 0.4 0.6 0.8
SMT_PFP_			**↑**	0.4 0.6										
PARITY (High → Low)													↓	0.2
TEAT_LENGTH_			**↑**	0.4 0.6	**↑**	0.4 0.6 0.8	**↑**	0.8	**↑**	0.6 0.8			**↑**	0.6
TEAT_DIAMETER_			**↑**	0.2										
YIELD	**↑**	0.8	**↑**	0.2	**↑**	0.2	**↑**	0.2 0.6	**↑**	0.2	**↑**	0.2	**↑**	0.2 0.8

‘up’ arrow corresponds to associated with increased ADOD and ‘down’ arrow corresponds to associated with decreased ADOD.
