# Supplementary material for: Evaluation of Two Practical Field Methods for Estimating Operational Overmilking Duration Using Standard Milking-System Sensors
Source: Animals (Basel). 2026 Jan 13;16(2):244. doi: 10.3390/ani16020244 (PMC12837506; doi:10.3390/ani16020244)
Supplement: Supplementary file 1 [file animals-16-00244-s001.zip › animals-4043526-supplementary.pdf]

Supplementary Materials

Supplementary Material S1: Front quarters (Q12)

Material S1.1: ADOD_VD_{OM}Q12_ACR_{OM}0.2:

The variables included in the initial ADOD_VD_{OM}Q12_ACR_{OM}0.2 models were as follows:

Equation S1:

ADOD_VD_{OM}Q12_ACR_{OM}0.2 = $HIGHFLOW_{TIME}$ + $LOWFLOW_{TIME}$ + $MACHINEON_{TIME}$ + MPC_{OM} + MPC_{PFP} + PARITY + $SLOWESTQ_{TIME}$ + SMT_{OM} + (AMF *OR* SMT_{TOT}) + YIELD ;

where $HIGHFLOW_{TIME}$ = period of high milk flow (seconds), $LOWFLOW_{TIME}$ = period of low milk flow (seconds), $MACHINEON_{TIME}$ = total milking time (seconds), MPC_{OM} = mouthpiece chamber vacuum during overmilking period (kPa), MPC_{PFP} = mouthpiece chamber vacuum during peak flow period (kPa), PARITY = parity, $SLOWESTQ_{TIME}$ = time associated with the slowest milking quarter (seconds), SMT_{OM} = short milk tube vacuum during the overmilking period (kPa), AMF = average milk flow rate (kg/min), SMT_{TOT} = short milk tube vacuum during total duration of milking (kPa), YIELD = milk yield (kg)

Material S1.2: ADOD_VD_{OM}Q12_ACR_{OM}0.4

The variables included in the initial ADOD_VD_{OM}Q12_ACR_{OM}0.4 models were as follows:

Equation S2:

ADOD_VD_{OM}Q12_ACR_{OM}0.4 = $HIGHFLOW_{TIME}$ + $LOWFLOW_{TIME}$ + $MACHINEON_{TIME}$ + MPC_{OM} + MPC_{PFP} + PARITY + $SLOWESTQ_{TIME}$ + SMT_{OM} + (AMF *OR* SMT_{TOT}) + YIELD ;

where $HIGHFLOW_{TIME}$ = period of high milk flow (seconds), $LOWFLOW_{TIME}$ = period of low milk flow (seconds), $MACHINEON_{TIME}$ = total milking time (seconds), MPC_{OM} = mouthpiece chamber vacuum during overmilking period (kPa), MPC_{PFP} = mouthpiece chamber vacuum during peak flow period (kPa), PARITY = parity, $SLOWESTQ_{TIME}$ = time associated with the slowest milking quarter (seconds), SMT_{OM} = short milk tube vacuum during the overmilking period (kPa), AMF = average milk flow rate (kg/min), SMT_{TOT} = short milk tube vacuum during total duration of milking (kPa), YIELD = milk yield (kg)

Material S1.3: ADOD_VD_{OM}Q12_ACR_{OM}0.6

The variables included in the initial ADOD_VD_{OM}Q12_ACR_{OM}0.6 models were as follows:

Equation S3:

$$\mathbf{ADOD\_VD_{OM}Q12\_ACR_{OM}0.6 = HIGHFLOW_{TIME} + LOWFLOW_{TIME} + MACHINEON_{TIME} + MPC_{OM} + MPC_{PFP} + PARITY + SLOWESTQ_{TIME} + SMT_{OM} + (AMF}\ \mathit{OR}\ \mathbf{SMT_{TOT}) + YIELD\ ;}$$

where $HIGHFLOW_{TIME}$ = period of high milk flow (seconds), $LOWFLOW_{TIME}$ = period of low milk flow (seconds), $MACHINEON_{TIME}$ = total milking time (seconds), MPC_{OM} = mouthpiece chamber vacuum during overmilking period (kPa), MPC_{PFP} = mouthpiece chamber vacuum during peak flow period (kPa), PARITY = parity, $SLOWESTQ_{TIME}$ = time associated with the slowest milking quarter (seconds), SMT_{OM} = short milk tube vacuum during the overmilking period (kPa), AMF = average milk flow rate (kg/min), SMT_{TOT} = short milk tube vacuum during total duration of milking (kPa), YIELD = milk yield (kg)

Material S1.4: $ADOD\_VD_{OM}Q12\_ACR_{OM}0.8$

The variables included in the initial $ADOD\_VD_{OM}Q12\_ACR_{OM}0.8$ models were as follows:

Equation S4:

$$\mathbf{ADOD\_VD_{OM}Q12\_ACR_{OM}0.8 = LOWFLOW_{TIME} + DEAD_{TIME} + MACHINEON_{TIME} + (MPC_{OM} + MPC_{PFP}}\ \mathit{OR}\ \mathbf{MPC_{TOT}) + PARITY + SLOWESTQ_{TIME} + SMT_{OM} + SMT_{TOT} + TEAT_{DIAMETER} + TEAT_{LENGTH} + YIELD\ ;}$$

where $LOWFLOW_{TIME}$ = period of low milk flow (seconds), DEADTIME = period of dead time (seconds), $MACHINEON_{TIME}$ = total milking time (seconds), MPC_{OM} = mouthpiece chamber vacuum during overmilking period (kPa), MPC_{PFP} = mouthpiece chamber vacuum during peak flow period (kPa), MPC_{TOT} = mouthpiece chamber vacuum during total duration of milking (kPa), PARITY = parity, $SLOWESTQ_{TIME}$ = time associated with the slowest milking quarter (seconds), SMT_{OM} = short milk tube vacuum during the overmilking period (kPa), SMT_{TOT} = short milk tube vacuum during total milking (kPa), $TEAT_{DIAMETER}$ = measurement of teat diameter (mm), $TEAT_{LENGTH}$ = measurement of teat length (mm), YIELD = milk yield (kg)

Supplementary Material S2: Rear quarters (Q34)

Material S2.1: $ADOD\_VD_{OM}Q34\_ACR_{OM}0.2$

The variables included in the initial $ADOD\_VD_{OM}Q34\_ACR_{OM}0.2$ models were as follows:

Equation S5:

$$\mathbf{ADOD\_VD_{OM}Q34\_ACR_{OM}0.2 = LOWFLOW_{TIME} + MACHINEON_{TIME} + (MPC_{OM} + MPC_{PFP}}\ \mathit{OR}\ \mathbf{MPC_{TOT}) + PARITY + SLOWESTQ_{TIME} + SMT_{OM} + SMT_{TOT} + TEAT_{DIAMETER} + TEAT_{LENGTH} + YIELD\ ;}$$

where $LOWFLOW_{TIME}$ = period of low milk flow (seconds), $MACHINEON_{TIME}$ = total milking time (seconds), MPC_{OM} = mouthpiece chamber vacuum during overmilking period (kPa), MPC_{PFP} = mouthpiece chamber vacuum during peak flow period (kPa), MPC_{TOT} = mouthpiece chamber vacuum during total duration of milking (kPa), PARITY = parity, $SLOWESTQ_{TIME}$ = time associated with the slowest milking quarter (seconds), SMT_{OM} = short milk tube vacuum during the overmilking period (kPa), SMT_{TOT} = short milk tube vacuum during total milking (kPa), $TEAT_{DIAMETER}$ = measurement of teat diameter (mm), $TEAT_{LENGTH}$ = measurement of teat length (mm), YIELD = milk yield (kg)

Material S2.2: ADOD_VD$_{OM}$Q34_ACR$_{OM}$0.4

The variables included in the initial ADOD_VD$_{OM}$Q34_ACR$_{OM}$0.4 models were as follows:

Equation S6:

ADOD_VD$_{OM}$Q34_ACR$_{OM}$0.4 = $LOWFLOW_{TIME}$ + (MPC_{OM} + MPC_{PFP} *OR* MPC_{TOT}) + PARITY + $SLOWESTQ_{TIME}$ + SMT_{OM} + SMT_{TOT} + $TEAT_{DIAMETER}$ + $TEAT_{LENGTH}$ + YIELD ;

where $LOWFLOW_{TIME}$ = period of low milk flow (seconds), MPC_{OM} = mouthpiece chamber vacuum during overmilking period (kPa), MPC_{PFP} = mouthpiece chamber vacuum during peak flow period (kPa), MPC_{TOT} = mouthpiece chamber vacuum during total duration of milking (kPa), PARITY = parity, $SLOWESTQ_{TIME}$ = time associated with the slowest milking quarter (seconds), SMT_{OM} = short milk tube vacuum during the overmilking period (kPa), SMT_{TOT} = short milk tube vacuum during total milking (kPa), $TEAT_{DIAMETER}$ = measurement of teat diameter (mm), $TEAT_{LENGTH}$ = measurement of teat length (mm), YIELD = milk yield (kg)

Material S2.3: ADOD_VD$_{OM}$Q34_ACR$_{OM}$0.6

The variables included in the initial ADOD_VD$_{OM}$Q34_ACR$_{OM}$0.6 models were as follows:

Equation S7:

ADOD_VD$_{OM}$Q34_ACR$_{OM}$0.6 = (MPC_{OM} + MPC_{PFP} *OR* MPC_{TOT}) + PARITY + $SLOWESTQ_{TIME}$ + SMT_{OM} + SMT_{TOT} + $TEAT_{DIAMETER}$ + $TEAT_{LENGTH}$ + YIELD ;

where MPC_{OM} = mouthpiece chamber vacuum during overmilking period (kPa), MPC_{PFP} = mouthpiece chamber vacuum during peak flow period (kPa), MPC_{TOT} = mouthpiece chamber vacuum during total duration of milking (kPa), PARITY = parity, $SLOWESTQ_{TIME}$ = time associated with the slowest milking quarter (seconds), SMT_{OM} = short milk tube vacuum during the overmilking period (kPa), SMT_{TOT} = short milk tube vacuum during total milking (kPa), $TEAT_{DIAMETER}$ = measurement of teat diameter (mm), $TEAT_{LENGTH}$ = measurement of teat length (mm), YIELD = milk yield (kg)

Material S2.4: ADOD_VD_{OM}Q34_ACR_{OM}0.8

The variables included in the initial ADOD_VD_{OM}Q34_ACR_{OM}0.8 models were as follows:

Equation S8:

ADOD_VD_{OM}Q34_ACR_{OM}0.8 = $HIGHFLOW_{TIME}$ + $MACHINEON_{TIME}$ + (MPC_{OM} + MPC_{PFP} *OR* MPC_{TOT}) + PARITY + $SLOWESTQ_{TIME}$ + SMT_{OM} + $TEAT_{DIAMETER}$;

where $HIGHFLOW_{TIME}$ = period of high milk flow (seconds), $MACHINEON_{TIME}$ = total milking time (seconds), MPC_{OM} = mouthpiece chamber vacuum during overmilking period (kPa), MPC_{PFP} = mouthpiece chamber vacuum during peak flow period (kPa), MPC_{TOT} = mouthpiece chamber vacuum during total duration of milking (kPa), PARITY = parity, $SLOWESTQ_{TIME}$ = time associated with the slowest milking quarter (seconds), SMT_{OM} = short milk tube vacuum during the overmilking period (kPa), $TEAT_{DIAMETER}$ = measurement of teat diameter (mm)

Supplementary Material S3: Left quarters (Q14)

Material S3.1: ADOD_VD_{OM}Q14_ACR_{OM}0.2

The variables included in the initial ADOD_VD_{OM}Q14_ACR_{OM}0.2 models were as follows:

Equation S9:

ADOD_VD_{OM}Q14_ACR_{OM}0.2 = $LOWFLOW_{TIME}$ + $DEAD_{TIME}$ + $MACHINEON_{TIME}$ + (MPC_{OM} + MPC_{PFP} *OR* MPC_{TOT}) + PARITY + $SLOWESTQ_{TIME}$ + SMT_{OM} + SMT_{TOT} + YIELD ;

where $LOWFLOW_{TIME}$ = period of low milk flow (seconds), DEADTIME = period of dead time (seconds), $MACHINEON_{TIME}$ = total milking time (seconds), MPC_{OM} = mouthpiece chamber vacuum during overmilking period (kPa), MPC_{PFP} = mouthpiece chamber vacuum during peak flow period (kPa), MPC_{TOT} = mouthpiece chamber vacuum during total duration of milking (kPa), PARITY = parity, $SLOWESTQ_{TIME}$ = time associated with the slowest milking quarter (seconds), SMT_{OM} = short milk tube vacuum during the overmilking period (kPa), SMT_{TOT} = short milk tube vacuum during total milking (kPa), YIELD = milk yield (kg)

Material S3.2: ADOD_VD_{OM}Q14_ACR_{OM}0.4

The variables included in the initial ADOD_VD_{OM}Q14_ACR_{OM}0.4 models were as follows:

Equation S10:

ADOD_VD_{OM}Q14_ACR_{OM}0.4 = $HIGHFLOW_{TIME}$ + $LOWFLOW_{TIME}$ + $MACHINEON_{TIME}$ + (MPC_{OM} + MPC_{PFP} *OR* MPC_{TOT}) + PARITY + $SLOWESTQ_{TIME}$ + SMT_{OM} + $TEAT_{LENGTH}$ + YIELD ;

where $HIGHFLOW_{TIME}$ = period of high milk flow (seconds), $LOWFLOW_{TIME}$ = period of low milk flow (seconds), $MACHINEON_{TIME}$ = total milking time (seconds), MPC_{OM} = mouthpiece chamber vacuum during overmilking period (kPa), MPC_{PFP} = mouthpiece chamber vacuum during peak flow period (kPa), MPC_{TOT} = mouthpiece chamber vacuum during total duration of milking (kPa), PARITY = parity, $SLOWESTQ_{TIME}$ = time associated with the slowest milking quarter (seconds), SMT_{OM} = short milk tube vacuum during the overmilking period (kPa), $TEAT_{LENGTH}$ = measurement of teat length (mm), YIELD = milk yield (kg)

Material S3.2: $ADOD\_VD_{OM}Q14\_ACR_{OM}0.6$

The variables included in the initial $ADOD\_VD_{OM}Q14\_ACR_{OM}0.6$ models were as follows:

Equation S11:

$$\mathbf{ADOD\_VD_{OM}Q14\_ACR_{OM}0.6 = HIGHFLOW_{TIME} + LOWFLOW_{TIME} + MACHINEON_{TIME} + (MPC_{OM} + MPC_{PFP}\ \mathit{OR}\ MPC_{TOT}) + PARITY + SLOWESTQ_{TIME} + SMT_{OM} + TEAT_{LENGTH} + YIELD\ ;}$$

where $HIGHFLOW_{TIME}$ = period of high milk flow (seconds), $LOWFLOW_{TIME}$ = period of low milk flow (seconds), $MACHINEON_{TIME}$ = total milking time (seconds), MPC_{OM} = mouthpiece chamber vacuum during overmilking period (kPa), MPC_{PFP} = mouthpiece chamber vacuum during peak flow period (kPa), MPC_{TOT} = mouthpiece chamber vacuum during total duration of milking (kPa), PARITY = parity, $SLOWESTQ_{TIME}$ = time associated with the slowest milking quarter (seconds), SMT_{OM} = short milk tube vacuum during the overmilking period (kPa), $TEAT_{LENGTH}$ = measurement of teat length (mm), YIELD = milk yield (kg)

Material S3.4: $ADOD\_VD_{OM}Q14\_ACR_{OM}0.8$

The variables included in the initial $ADOD\_VD_{OM}Q14\_ACR_{OM}0.8$ models were as follows:

Equation S12:

$$\mathbf{ADOD\_VD_{OM}Q14\_ACR_{OM}0.8 = HIGHFLOW_{TIME} + LOWFLOW_{TIME} + MACHINEON_{TIME} + (MPC_{OM} + MPC_{PFP}\ \mathit{OR}\ MPC_{TOT}) + PARITY + SLOWESTQ + SLOWESTQ_{TIME} + SMT_{OM} + TEAT_{LENGTH} + YIELD\ ;}$$

where $HIGHFLOW_{TIME}$ = period of high milk flow (seconds), $LOWFLOW_{TIME}$ = period of low milk flow (seconds), $MACHINEON_{TIME}$ = total milking time (seconds), MPC_{OM} = mouthpiece chamber vacuum during overmilking period (kPa), MPC_{PFP} = mouthpiece chamber vacuum during peak flow period (kPa), MPC_{TOT} = mouthpiece chamber vacuum during total duration of milking (kPa), PARITY = parity, $SLOWESTQ_{TIME}$ = time associated with the slowest milking quarter (seconds),

SMT_{OM} = short milk tube vacuum during the overmilking period (kPa), $TEAT_{LENGTH}$ = measurement of teat length (mm), YIELD = milk yield (kg)

Supplementary Material S4: Right quarters (Q23)

Material S4.1: $ADOD\_VD_{OM}Q23\_ACR_{OM}0.2$

The variables included in the initial $ADOD\_VD_{OM}Q23\_ACR_{OM}0.2$ models were as follows:

Equation S13:

$$\mathbf{ADOD\_VD_{OM}Q23\_ACR_{OM}0.2 = LOWFLOW_{TIME} + MACHINEON_{TIME} + (MPC_{OM}} \textit{ OR } \mathbf{MPC_{PFP}} \textit{ OR } \mathbf{MPC_{TOT}) + PARITY + LOGSCC + SLOWESTQ + SLOWESTQ_{TIME} + SMT_{OM} + SMT_{TOT} + TEAT_{DIAMETER} + YIELD\ ;}$$

where $LOWFLOW_{TIME}$ = period of low milk flow (seconds), $MACHINEON_{TIME}$ = total milking time (seconds), MPC_{OM} = mouthpiece chamber vacuum during overmilking period (kPa), MPC_{PFP} = mouthpiece chamber vacuum during peak flow period (kPa), MPC_{TOT} = mouthpiece chamber vacuum during total duration of milking (kPa), PARITY = parity, LOGSCC = logarithmic-10 transformed somatic cell count (cells/ml), SLOWESTQ = slowest milking quarter, $SLOWESTQ_{TIME}$ = time associated with the slowest milking quarter (seconds), SMT_{OM} = short milk tube vacuum during the overmilking period (kPa), SMT_{TOT} = short milk tube vacuum during total milking (kPa), $TEAT_{DIAMETER}$ = measurement of teat diameter (mm), YIELD = milk yield (kg)

Material S4.2: $ADOD\_VD_{OM}Q23\_ACR_{OM}0.4$

The variables included in the initial $ADOD\_VD_{OM}Q23\_ACR_{OM}0.4$ models were as follows:

Equation S14:

$$\mathbf{ADOD\_VD_{OM}Q23\_ACR_{OM}0.4 = HIGHFLOW_{TIME} + LOWFLOW_{TIME} + MACHINEON_{TIME} + (MPC_{OM}} \textit{ OR } \mathbf{MPC_{PFP}} \textit{ OR } \mathbf{MPC_{TOT}) + PARITY + LOGSCC + SLOWESTQ + SLOWESTQ_{TIME} + SMT_{OM} + TEAT_{DIAMETER} + YIELD\ ;}$$

where $HIGHFLOW_{TIME}$ = period of high milk flow (seconds), $LOWFLOW_{TIME}$ = period of low milk flow (seconds), $MACHINEON_{TIME}$ = total milking time (seconds), MPC_{OM} = mouthpiece chamber vacuum during overmilking period (kPa), MPC_{PFP} = mouthpiece chamber vacuum during peak flow period (kPa), MPC_{TOT} = mouthpiece chamber vacuum during total duration of milking (kPa), PARITY = parity, LOGSCC = logarithmic-10 transformed somatic cell count (cells/ml), SLOWESTQ = slowest milking quarter, $SLOWESTQ_{TIME}$ = time associated with the slowest milking quarter (seconds), SMT_{OM} = short milk tube vacuum during the overmilking period (kPa), $TEAT_{DIAMETER}$ = measurement of teat diameter (mm), YIELD = milk yield (kg)

Material S4.3: ADOD_VD$_{OM}$Q23_ACR$_{OM}$0.6

The variables included in the initial ADOD_VD$_{OM}$Q23_ACR$_{OM}$0.6 models were as follows:

Equation S15:

ADOD_VD$_{OM}$Q23_ACR$_{OM}$0.6 = HIGHFLOW$_{TIME}$ + LOWFLOW$_{TIME}$ + MACHINEON$_{TIME}$ + (MPC$_{OM}$ *OR* MPC$_{PFP}$ *OR* MPC$_{TOT}$) + PARITY + SLOWESTQ + SLOWESTQ$_{TIME}$ + SMT$_{OM}$ + TEAT$_{DIAMETER}$ + TEAT$_{LENGTH}$ + YIELD ;

where HIGHFLOW$_{TIME}$ = period of high milk flow (seconds), LOWFLOW$_{TIME}$ = period of low milk flow (seconds), MACHINEON$_{TIME}$ = total milking time (seconds), MPC$_{OM}$ = mouthpiece chamber vacuum during overmilking period (kPa), MPC$_{PFP}$ = mouthpiece chamber vacuum during peak flow period (kPa), MPC$_{TOT}$ = mouthpiece chamber vacuum during total duration of milking (kPa), PARITY = parity, SLOWESTQ = slowest milking quarter, SLOWESTQ$_{TIME}$ = time associated with the slowest milking quarter (seconds), SMT$_{OM}$ = short milk tube vacuum during the overmilking period (kPa), TEAT$_{DIAMETER}$ = measurement of teat diameter (mm), TEAT$_{LENGTH}$ = measurement of teat length (mm), YIELD = milk yield (kg)

Material S4.4: ADOD_VD$_{OM}$Q23_ACR$_{OM}$0.8

The variables included in the initial ADOD_VD$_{OM}$Q23_ACR$_{OM}$0.8 models were as follows:

Equation S16:

ADOD_VD$_{OM}$Q23_ACR$_{OM}$0.8 = HIGHFLOW$_{TIME}$ + LOWFLOW$_{TIME}$ + MACHINEON$_{TIME}$ + (MPC$_{OM}$ *OR* MPC$_{PFP}$ *OR* MPC$_{TOT}$) + PARITY + SLOWESTQ$_{TIME}$ + SMT$_{OM}$ + TEAT$_{DIAMETER}$ + TEAT$_{LENGTH}$ + YIELD ;

where HIGHFLOW$_{TIME}$ = period of high milk flow (seconds), LOWFLOW$_{TIME}$ = period of low milk flow (seconds), MACHINEON$_{TIME}$ = total milking time (seconds), MPC$_{OM}$ = mouthpiece chamber vacuum during overmilking period (kPa), MPC$_{PFP}$ = mouthpiece chamber vacuum during peak flow period (kPa), MPC$_{TOT}$ = mouthpiece chamber vacuum during total duration of milking (kPa), PARITY = parity, SLOWESTQ$_{TIME}$ = time associated with the slowest milking quarter (seconds), SMT$_{OM}$ = short milk tube vacuum during the overmilking period (kPa), TEAT$_{DIAMETER}$ = measurement of teat diameter (mm), TEAT$_{LENGTH}$ = measurement of teat length (mm), YIELD = milk yield (kg)

Supplementary Material S5: Front right and rear left quarters (Q24)

Material S5.1: ADOD_VD$_{OM}$Q24_ACR$_{OM}$0.2

The variables included in the initial ADOD_VD$_{OM}$Q24_ACR$_{OM}$0.2 models were as follows:

Equation S17:

$$\textbf{ADOD\_VD}_{\textbf{OM}}\textbf{Q24\_ACR}_{\textbf{OM}}\textbf{0.2} = \textbf{LOWFLOW}_{\textbf{TIME}} + \textbf{DEAD}_{\textbf{TIME}} + \textbf{MACHINEON}_{\textbf{TIME}} + (\textbf{MPC}_{\textbf{OM}}\ \textit{OR}\ \textbf{MPC}_{\textbf{PFP}}\ \textit{OR}\ \textbf{MPC}_{\textbf{TOT}}) + \textbf{PARITY} + \textbf{SLOWESTQ}_{\textbf{TIME}} + \textbf{SMT}_{\textbf{OM}} + \textbf{SMT}_{\textbf{TOT}} + \textbf{TEAT}_{\textbf{DIAMETER}} + \textbf{TEAT}_{\textbf{LENGTH}} + \textbf{YIELD}\ ;$$

where $LOWFLOW_{TIME}$ = period of low milk flow (seconds), $DEAD_{TIME}$ = period of dead time (seconds), $MACHINEON_{TIME}$ = total milking time (seconds), MPC_{OM} = mouthpiece chamber vacuum during overmilking period (kPa), MPC_{PFP} = mouthpiece chamber vacuum during peak flow period (kPa), MPC_{TOT} = mouthpiece chamber vacuum during total duration of milking (kPa), PARITY = parity, $SLOWESTQ_{TIME}$ = time associated with the slowest milking quarter (seconds), SMT_{OM} = short milk tube vacuum during the overmilking period (kPa), SMT_{TOT} = short milk tube vacuum during total milking (kPa), $TEAT_{DIAMETER}$ = measurement of teat diameter (mm), $TEAT_{LENGTH}$ = measurement of teat length (mm), YIELD = milk yield (kg)

Material S5.2: ADOD_VD_{OM}Q24_ACR_{OM}0.4

The variables included in the initial ADOD_VD_{OM}Q24_ACR_{OM}0.4 models were as follows:

Equation S18:

$$\textbf{ADOD\_VD}_{\textbf{OM}}\textbf{Q24\_ACR}_{\textbf{OM}}\textbf{0.4} = \textbf{LOWFLOW}_{\textbf{TIME}} + \textbf{MACHINEON}_{\textbf{TIME}} + (\textbf{MPC}_{\textbf{OM}}\ \textit{OR}\ \textbf{MPC}_{\textbf{PFP}}\ \textit{OR}\ \textbf{MPC}_{\textbf{TOT}}) + \textbf{PARITY} + \textbf{SLOWESTQ} + \textbf{SLOWESTQ}_{\textbf{TIME}} + \textbf{SMT}_{\textbf{OM}} + \textbf{TEAT}_{\textbf{DIAMETER}} + \textbf{TEAT}_{\textbf{LENGTH}} + \textbf{YIELD}\ ;$$

where $LOWFLOW_{TIME}$ = period of low milk flow (seconds), $MACHINEON_{TIME}$ = total milking time (seconds), MPC_{OM} = mouthpiece chamber vacuum during overmilking period (kPa), MPC_{PFP} = mouthpiece chamber vacuum during peak flow period (kPa), MPC_{TOT} = mouthpiece chamber vacuum during total duration of milking (kPa), PARITY = parity, SLOWESTQ = slowest milking quarter, $SLOWESTQ_{TIME}$ = time associated with the slowest milking quarter (seconds), SMT_{OM} = short milk tube vacuum during the overmilking period (kPa), $TEAT_{DIAMETER}$ = measurement of teat diameter (mm), $TEAT_{LENGTH}$ = measurement of teat length (mm), YIELD = milk yield (kg)

Material S5.3: ADOD_VD_{OM}Q24_ACR_{OM}0.6

The variables included in the initial ADOD_VD_{OM}Q24_ACR_{OM}0.6 models were as follows:

Equation S19:

$$\textbf{ADOD\_VD}_{\textbf{OM}}\textbf{Q24\_ACR}_{\textbf{OM}}\textbf{0.6} = \textbf{HIGHFLOW}_{\textbf{TIME}} + \textbf{LOWFLOW}_{\textbf{TIME}} + \textbf{MACHINEON}_{\textbf{TIME}} + (\textbf{MPC}_{\textbf{OM}}\ \textit{OR}\ \textbf{MPC}_{\textbf{PFP}}\ \textit{OR}\ \textbf{MPC}_{\textbf{TOT}}) + \textbf{PARITY} + \textbf{SLOWESTQ}_{\textbf{TIME}} + \textbf{SMT}_{\textbf{OM}} + \textbf{TEAT}_{\textbf{DIAMETER}} + \textbf{TEAT}_{\textbf{LENGTH}} + \textbf{YIELD}\ ;$$

where $HIGHFLOW_{TIME}$ = period of high milk flow (seconds), $LOWFLOW_{TIME}$ = period of low milk flow (seconds), $MACHINEON_{TIME}$ = total milking time (seconds), MPC_{OM} = mouthpiece chamber vacuum during overmilking period (kPa), MPC_{PFP} = mouthpiece chamber vacuum during peak flow period (kPa), MPC_{TOT} = mouthpiece chamber vacuum during total duration of milking (kPa), PARITY = parity, $SLOWESTQ_{TIME}$ = time associated with the slowest milking quarter (seconds), SMT_{OM} = short milk tube vacuum during the overmilking period (kPa), $TEAT_{DIAMETER}$ = measurement of teat diameter (mm), $TEAT_{LENGTH}$ = measurement of teat length (mm), YIELD = milk yield (kg)

Material S5.4: ADOD_VD_{OM}Q24_ACR_{OM}0.8

The variables included in the initial ADOD_VD_{OM}Q24_ACR_{OM}0.8 models were as follows:

Equation S20:

ADOD_VD_{OM}Q24_ACR_{OM}0.8 = $HIGHFLOW_{TIME}$ + $MACHINEON_{TIME}$ + (MPC_{OM} *OR* MPC_{PFP} *OR* MPC_{TOT}) + PARITY + $SLOWESTQ_{TIME}$ + SMT_{OM} + $TEAT_{DIAMETER}$ + $TEAT_{LENGTH}$ + $TB_{CONGESTION}$ + $TE_{CONGESTION}$ + YIELD ;

where $HIGHFLOW_{TIME}$ = period of high milk flow (seconds), $MACHINEON_{TIME}$ = total milking time (seconds), MPC_{OM} = mouthpiece chamber vacuum during overmilking period (kPa), MPC_{PFP} = mouthpiece chamber vacuum during peak flow period (kPa), MPC_{TOT} = mouthpiece chamber vacuum during total duration of milking (kPa), PARITY = parity, $SLOWESTQ_{TIME}$ = time associated with the slowest milking quarter (seconds), SMT_{OM} = short milk tube vacuum during the overmilking period (kPa), $TEAT_{DIAMETER}$ = measurement of teat diameter (mm), $TEAT_{LENGTH}$ = measurement of teat length (mm), $TB_{CONGESTION}$ = teat barrel congestion score, $TE_{CONGESTION}$ = teat end congestion score, YIELD = milk yield (kg)

Supplementary Material S6: Front left and rear right quarters (Q13)

Material S6.1: ADOD_VD_{OM}Q13_ACR_{OM}0.2

The variables included in the initial ADOD_VD_{OM}Q13_ACR_{OM}0.2 models were as follows:

Equation S21:

ADOD_VD_{OM}Q13_ACR_{OM}0.2 = $LOWFLOW_{TIME}$ + $MACHINEON_{TIME}$ + (MPC_{OM} + MPC_{PFP} *OR* MPC_{TOT}) + PARITY + SLOWESTQ + $SLOWESTQ_{TIME}$ + SMT_{OM} + SMT_{TOT} + YIELD ;

where $LOWFLOW_{TIME}$ = period of low milk flow (seconds), $MACHINEON_{TIME}$ = total milking time (seconds), MPC_{OM} = mouthpiece chamber vacuum during overmilking period (kPa), MPC_{PFP} = mouthpiece chamber vacuum during peak flow period (kPa), MPC_{TOT} = mouthpiece chamber vacuum during total duration of milking (kPa), PARITY = parity, SLOWESTQ = slowest milking quarter,

$SLOWESTQ_{TIME}$ = time associated with the slowest milking quarter (seconds), SMT_{OM} = short milk tube vacuum during the overmilking period (kPa), SMT_{TOT} = short milk tube vacuum during total milking (kPa), YIELD = milk yield (kg)

Material S6.2: $ADOD\_VD_{OM}Q13\_ACR_{OM}0.4$

The variables included in the initial $ADOD\_VD_{OM}Q13\_ACR_{OM}0.4$ models were as follows:

Equation S22:

$$\mathbf{ADOD\_VD_{OM}Q13\_ACR_{OM}0.4 = HIGHFLOW_{TIME} + LOWFLOW_{TIME} + DEAD_{TIME} + MACHINEON_{TIME} + (MPC_{OM} + MPC_{PFP}\ \mathit{OR}\ MPC_{TOT}) + PARITY + SLOWESTQ + SMT_{OM} + TB_{CONGESTION} + YIELD\ ;}$$

where $HIGHFLOW_{TIME}$ = period of high milk flow (seconds), $LOWFLOW_{TIME}$ = period of low milk flow (seconds), $DEAD_{TIME}$ = period of dead time (seconds), $MACHINEON_{TIME}$ = total milking time (seconds), MPC_{OM} = mouthpiece chamber vacuum during overmilking period (kPa), MPC_{PFP} = mouthpiece chamber vacuum during peak flow period (kPa), MPC_{TOT} = mouthpiece chamber vacuum during total duration of milking (kPa), PARITY = parity, SLOWESTQ = slowest milking quarter, SMT_{OM} = short milk tube vacuum during the overmilking period (kPa), $TB_{CONGESTION}$ = teat barrel congestion score, YIELD = milk yield (kg)

Material S6.3: $ADOD\_VD_{OM}Q13\_ACR_{OM}0.6$

The variables included in the initial $ADOD\_VD_{OM}Q13\_ACR_{OM}0.6$ models were as follows:

Equation S23:

$$\mathbf{ADOD\_VD_{OM}Q13\_ACR_{OM}0.6 = HIGHFLOW_{TIME} + LOWFLOW_{TIME} + DEAD_{TIME} + MACHINEON_{TIME} + (MPC_{OM} + MPC_{PFP}\ \mathit{OR}\ MPC_{TOT}) + PARITY + SLOWESTQ + SMT_{OM} + TEAT_{LENGTH} + YIELD\ ;}$$

where $HIGHFLOW_{TIME}$ = period of high milk flow (seconds), $LOWFLOW_{TIME}$ = period of low milk flow (seconds), $DEAD_{TIME}$ = period of dead time (seconds), $MACHINEON_{TIME}$ = total milking time (seconds), MPC_{OM} = mouthpiece chamber vacuum during overmilking period (kPa), MPC_{PFP} = mouthpiece chamber vacuum during peak flow period (kPa), MPC_{TOT} = mouthpiece chamber vacuum during total duration of milking (kPa), PARITY = parity, SLOWESTQ = slowest milking quarter, SMT_{OM} = short milk tube vacuum during the overmilking period (kPa), $TEAT_{LENGTH}$ = measurement of teat length (mm), YIELD = milk yield (kg)

Material S6.4: $ADOD\_VD_{OM}Q13\_ACR_{OM}0.8$

The variables included in the initial $ADOD\_VD_{OM}Q13\_ACR_{OM}0.8$ models were as follows:

Equation S24:

$$\mathbf{ADOD\_VD_{OM}Q13\_ACR_{OM}0.8 = HIGHFLOW_{TIME} + LOWFLOW_{TIME} + DEAD_{TIME} + MACHINEON_{TIME} + (MPC_{OM} + MPC_{PFP}}\ \mathit{OR}\ \mathbf{MPC_{TOT}) + PARITY + SLOWESTQ + SMT_{OM} + YIELD\ ;}$$

where $HIGHFLOW_{TIME}$ = period of high milk flow (seconds), $LOWFLOW_{TIME}$ = period of low milk flow (seconds), $DEAD_{TIME}$ = period of dead time (seconds), $MACHINEON_{TIME}$ = total milking time (seconds), MPC_{OM} = mouthpiece chamber vacuum during overmilking period (kPa), MPC_{PFP} = mouthpiece chamber vacuum during peak flow period (kPa), MPC_{TOT} = mouthpiece chamber vacuum during total duration of milking (kPa), PARITY = parity, SLOWESTQ = slowest milking quarter, SMT_{OM} = short milk tube vacuum during the overmilking period (kPa), YIELD = milk yield (kg)

Supplementary Material S7: All four quarters (Q1234)

Material S7.1: $ADOD\_VD_{OM}Q1234\_ACR_{OM}0.2$

The variables included in the initial $ADOD\_VD_{OM}Q1234\_ACR_{OM}0.2$ models were as follows:

Equation S25:

$$\mathbf{ADOD\_VD_{OM}Q1234\_ACR_{OM}0.2 = LOWFLOW_{TIME} + MACHINEON_{TIME} + (MPC_{OM}}\ \mathit{OR}\ \mathbf{MPC_{PFP}}\ \mathit{OR}\ \mathbf{MPC_{TOT}) + PARITY + SLOWESTQ_{TIME} + SMT_{OM} + SMT_{TOT} + TEAT_{DIAMETER} + TEAT_{LENGTH} + YIELD\ ;}$$

where $LOWFLOW_{TIME}$ = period of low milk flow (seconds), $MACHINEON_{TIME}$ = total milking time (seconds), MPC_{OM} = mouthpiece chamber vacuum during overmilking period (kPa), MPC_{PFP} = mouthpiece chamber vacuum during peak flow period (kPa), MPC_{TOT} = mouthpiece chamber vacuum during total duration of milking (kPa), PARITY = parity, $SLOWESTQ_{TIME}$ = time associated with the slowest milking quarter (seconds), SMT_{OM} = short milk tube vacuum during the overmilking period (kPa), SMT_{TOT} = short milk tube vacuum during total milking (kPa), $TEAT_{DIAMETER}$ = measurement of teat diameter (mm), $TEAT_{LENGTH}$ = measurement of teat length (mm), YIELD = milk yield (kg)

Material S7.2: $ADOD\_VD_{OM}Q1234\_ACR_{OM}0.4$

The variables included in the initial $ADOD\_VD_{OM}Q1234\_ACR_{OM}0.4$ models were as follows:

Equation S26:

$ADOD\_VD_{OM}Q1234\_ACR_{OM}0.4 = HIGHFLOW_{TIME} + LOWFLOW_{TIME} + MACHINEON_{TIME} + (MPC_{OM}$ *OR* MPC_{PFP} *OR* $MPC_{TOT}) + PARITY + SLOWESTQ + SLOWESTQ_{TIME} + SMT_{OM} + SMT_{TOT} + TEAT_{LENGTH} + YIELD$;

where $HIGHFLOW_{TIME}$ = period of high milk flow (seconds), $LOWFLOW_{TIME}$ = period of low milk flow (seconds), $MACHINEON_{TIME}$ = total milking time (seconds), MPC_{OM} = mouthpiece chamber vacuum during overmilking period (kPa), MPC_{PFP} = mouthpiece chamber vacuum during peak flow period (kPa), MPC_{TOT} = mouthpiece chamber vacuum during total duration of milking (kPa), PARITY = parity, SLOWESTQ = slowest milking quarter, $SLOWESTQ_{TIME}$ = time associated with the slowest milking quarter (seconds), SMT_{OM} = short milk tube vacuum during the overmilking period (kPa), SMT_{TOT} = short milk tube vacuum during total milking (kPa), $TEAT_{LENGTH}$ = measurement of teat length (mm), YIELD = milk yield (kg)

Material S7.3: $ADOD\_VD_{OM}Q1234\_ACR_{OM}0.6$

The variables included in the initial $ADOD\_VD_{OM}Q1234\_ACR_{OM}0.6$ models were as follows:

Equation S27:

$ADOD\_VD_{OM}Q1234\_ACR_{OM}0.6 = HIGHFLOW_{TIME} + LOWFLOW_{TIME} + MACHINEON_{TIME} + (MPC_{OM}$ *OR* MPC_{PFP} *OR* $MPC_{TOT}) + PARITY + SLOWESTQ + SLOWESTQ_{TIME} + SMT_{OM} + TEAT_{DIAMETER} + TEAT_{LENGTH} + TE_{CONGESTION} + YIELD$;

where $HIGHFLOW_{TIME}$ = period of high milk flow (seconds), $LOWFLOW_{TIME}$ = period of low milk flow (seconds), $MACHINEON_{TIME}$ = total milking time (seconds), MPC_{OM} = mouthpiece chamber vacuum during overmilking period (kPa), MPC_{PFP} = mouthpiece chamber vacuum during peak flow period (kPa), MPC_{TOT} = mouthpiece chamber vacuum during total duration of milking (kPa), PARITY = parity, SLOWESTQ = slowest milking quarter, $SLOWESTQ_{TIME}$ = time associated with the slowest milking quarter (seconds), SMT_{OM} = short milk tube vacuum during the overmilking period (kPa), $TEAT_{DIAMETER}$ = measurement of teat diameter (mm), $TEAT_{LENGTH}$ = measurement of teat length (mm), $TE_{CONGESTION}$ = teat end congestion score, YIELD = milk yield (kg)

Material S7.4: $ADOD\_VD_{OM}Q1234\_ACR_{OM}0.8$

The variables included in the initial $ADOD\_VD_{OM}Q1234\_ACR_{OM}0.8$ models were as follows:

Equation S28:

$$\mathbf{ADOD\_VD_{OM}Q1234\_ACR_{OM}0.8 = LOWFLOW_{TIME} + DEAD_{TIME} + MACHINEON_{TIME} + (MPC_{OM}}\ \mathit{OR}\ \mathbf{MPC_{PFP}}\ \mathit{OR}\ \mathbf{MPC_{TOT}) + PARITY + SLOWESTQ + SLOWESTQ_{TIME} + SMT_{OM} + SMT_{TOT} + TEAT_{DIAMETER} + TEAT_{LENGTH} + YIELD}\ ;$$

where $LOWFLOW_{TIME}$ = period of low milk flow (seconds), $DEAD_{TIME}$ = period of dead time (seconds), $MACHINEON_{TIME}$ = total milking time (seconds), MPC_{OM} = mouthpiece chamber vacuum during overmilking period (kPa), MPC_{PFP} = mouthpiece chamber vacuum during peak flow period (kPa), MPC_{TOT} = mouthpiece chamber vacuum during total duration of milking (kPa), PARITY = parity, SLOWESTQ = slowest milking quarter, $SLOWESTQ_{TIME}$ = time associated with the slowest milking quarter (seconds), SMT_{OM} = short milk tube vacuum during the overmilking period (kPa), SMT_{TOT} = short milk tube vacuum during total milking (kPa), $TEAT_{DIAMETER}$ = measurement of teat diameter (mm), $TEAT_{LENGTH}$ = measurement of teat length (mm), YIELD = milk yield (kg)

Supplementary Tables

Supplementary Table S1: Investigation of the factors influencing the first (Qt1) and fourth (Qt4) quartiles of ADOD for the front quarters (Q12) at simulated ACR take-off thresholds of 0.2-0.8 kg/min

**FRONT QUARTERS (Q12)**								
	**0.2**		**0.4**		**0.6**		**0.8**	
	Qt1	Qt4	Qt1	Qt4	Qt1	Qt4	Qt1	Qt4
Front quarter = SLOWESTQ	-	-	-	-	-	-	-	-
Rear quarter = SLOWESTQ	-	-	-	-	-	-	-	-
Front SLOWESTQ$_{TIME}$ same as rear	19.23%	0.00%	19.23%	0.00%	17.86%	0.00%	15.38%	0.00%
SLOWESTQ poor MPC vacuum phase contrast	38.46%	12.00%	42.31%	12.00%	35.71%	7.69%	34.62%	7.69%
TEAT$_{DIAMETER}$ of SLOWESTQ (mm)	29.60	30.65	29.38	30.00	30.00	30.00	30.22	30.21
PARITY	1.85	2.72	1.69	2.68	1.79	3.12	1.73	2.88
DIM	32.81	30.48	34.92	32.40	31.43	32.46	30.08	31.58
YIELD (kg)	14.22	17.55	14.33	17.69	14.60	18.37	14.72	18.30
MACHINEON$_{TIME}$ (s)	393.38	577.08	431.54	597.08	425.57	577.85	443.85	565.46
VD$_{OM}$ (s)	79.35	243.44	80.81	242.76	81.18	243.50	85.69	240.62
LOWFLOW$_{TIME}$ (s)	92.00	193.50	109.60	199.67	119.25	180.19	126.27	170.15
HIGHFLOW$_{TIME}$ (s)	270.85	329.76	269.58	345.84	274.82	363.54	285.19	361.08
DEAD$_{TIME}$ (s)	2.69	4.20	3.08	4.40	3.21	4.62	2.88	4.42
SMT$_{TOT}$ (kPa)	35.36	36.33	35.83	36.58	35.78	36.25	36.03	36.13
SMT$_{PFP}$ (kPa)	34.04	34.32	34.62	34.98	34.45	34.39	34.75	34.23
SMT$_{OM}$ (kPa)	40.81	39.18	41.21	39.08	41.26	38.73	41.33	38.77
MPC$_{TOT}$ (kPa)	30.49	26.67	31.98	27.13	30.44	26.13	29.90	26.14
MPC$_{PFP}$ (kPa)	28.94	21.50	31.00	22.27	29.08	20.52	28.57	20.74
MPC$_{OM}$ (kPa)	36.09	32.80	35.98	33.81	35.32	33.10	35.14	32.62
ADOD_VD$_{OM}$Q12_ACR$_{OM}$	69.94	258.96	46.46	229.10	41.73	207.33	29.02	189.21

Supplementary Table S2: Investigation of the factors influencing the first (Qt1) and fourth (Qt4) quartiles of ADOD for the rear quarters (Q34) at simulated ACR take-off thresholds of 0.2-0.8 kg/min

REAR QUARTERS (Q34)								
	**0.2**		**0.4**		**0.6**		**0.8**	
	Qt1	Qt4	Qt1	Qt4	Qt1	Qt4	Qt1	Qt4
Front quarter = SLOWESTQ	-	-	-	-	-	-	-	-
Rear quarter = SLOWESTQ	-	-	-	-	-	-	-	-
Front SLOWESTQ$_{TIME}$ same as rear	23.08%	0.00%	19.23%	3.85%	19.23%	3.85%	14.81%	3.85%
SLOWESTQ poor MPC vacuum phase contrast	23.08%	4.00%	23.08%	15.38%	26.92%	11.54%	22.22%	11.54%
TEAT$_{DIAMETER}$ of SLOWESTQ (mm)	28.80	31.74	29.78	30.71	29.09	31.80	30.00	31.25
PARITY	2.19	3.28	1.81	3.15	1.81	3.46	2.19	3.04
DIM	34.27	30.28	32.65	29.69	29.85	34.31	30.96	32.35
YIELD (kg)	15.50	18.41	16.15	18.12	16.25	17.99	16.00	17.37
MACHINEON$_{TIME}$ (s)	410.42	524.84	502.58	536.54	516.00	528.15	488.56	543.88
VD$_{OM}$ (s)	55.12	131.76	59.77	137.77	63.23	127.62	66.59	132.38
LOWFLOW$_{TIME}$ (s)	85.38	175.76	121.38	178.38	162.35	160.44	133.15	178.13
HIGHFLOW$_{TIME}$ (s)	300.73	318.80	312.77	328.42	325.38	321.00	328.22	298.77
DEAD$_{TIME}$ (s)	3.65	4.20	3.85	4.23	4.81	4.04	4.07	4.81
SMT$_{TOT}$ (kPa)	35.09	35.78	36.05	36.03	36.40	35.98	36.26	36.22
SMT$_{PFP}$ (kPa)	34.07	33.83	35.17	34.09	35.51	34.15	35.23	34.37
SMT$_{OM}$ (kPa)	41.18	40.72	41.88	40.63	42.03	40.71	41.94	40.67
MPC$_{TOT}$ (kPa)	31.36	24.68	32.33	25.15	32.51	24.33	31.37	25.50
MPC$_{PFP}$ (kPa)	30.47	21.70	31.58	22.14	31.75	21.48	30.43	22.56
MPC$_{OM}$ (kPa)	36.40	32.04	36.85	32.02	36.86	31.55	36.33	32.20
ADOD_VD$_{OM}$Q34_ACR$_{OM}$	54.24	172.45	20.08	121.56	13.38	101.48	9.09	90.19

Supplementary Table S3: Investigation of the factors influencing the first (Qt1) and fourth (Qt4) quartiles of ADOD for the left-hand-side quarters (Q14) at simulated ACR take-off thresholds of 0.2-0.8 kg/min

LEFT QUARTERS (Q14)								
	0.2		0.4		0.6		0.8	
	Qt1	Qt4	Qt1	Qt4	Qt1	Qt4	Qt1	Qt4
Front quarter = SLOWESTQ	38.46%	30.77%	50.00%	26.92%	50.00%	26.92%	42.31%	19.23%
Rear quarter = SLOWESTQ	61.54%	69.23%	50.00%	73.08%	50.00%	73.08%	57.69%	80.77%
Front SLOWESTQTIME same as rear	19.23%	3.85%	19.23%	3.85%	19.23%	3.85%	11.54%	0.00%
SLOWESTQ poor MPC vacuum phase contrast	30.77%	11.54%	38.46%	7.69%	38.46%	7.69%	34.62%	11.54%
TEATDIAMETER of SLOWESTQ (mm)	29.79	30.40	30.65	30.80	30.42	30.83	29.58	29.78
PARITY	1.88	3.23	1.77	3.42	1.73	3.35	1.88	2.96
DIM	35.58	27.81	34.50	31.27	34.27	30.58	31.77	29.69
YIELD (kg)	14.83	18.23	14.52	18.42	14.37	18.49	14.38	18.10
MACHINEONTIME (s)	407.38	569.88	429.62	566.96	417.50	569.23	443.12	568.15
VDOM (s)	60.00	150.31	75.46	152.65	76.08	149.92	71.65	132.08
LOWFLOWTIME (s)	99.50	208.27	104.32	200.19	107.77	198.81	136.42	186.80
HIGHFLOWTIME (s)	283.15	331.00	275.00	336.50	280.50	340.77	279.27	334.00
DEADTIME (s)	4.23	4.04	2.88	3.85	2.88	3.85	3.46	5.96
SMTTOT (kPa)	35.47	36.21	35.90	36.17	35.88	36.17	36.23	36.15
SMTPFP (kPa)	34.40	34.37	34.69	34.34	34.63	34.36	35.11	34.52
SMTOM (kPa)	41.22	40.32	41.68	40.19	41.64	40.25	41.85	40.40
MPCTOT (kPa)	30.62	24.05	30.38	23.28	30.32	23.19	31.60	26.23
MPCPFP (kPa)	29.63	20.69	29.36	19.87	29.27	19.84	30.74	23.47
MPCOM (kPa)	35.97	31.23	35.77	30.66	35.64	30.80	36.17	32.81
ADOD_VDOMQ14_ACROM	60.75	203.31	38.13	166.96	28.87	148.42	17.60	130.04

Supplementary Table S4: Investigation of the factors influencing the first (Qt1) and fourth (Qt4) quartiles of ADOD for the right-hand-side quarters (Q23) at simulated ACR take-off thresholds of 0.2-0.8 kg/min

**RIGHT QUARTERS (Q23)**								
	**0.2**		**0.4**		**0.6**		**0.8**	
	Qt1	Qt4	Qt1	Qt4	Qt1	Qt4	Qt1	Qt4
Front quarter = SLOWESTQ	57.69%	38.46%	61.54%	34.62%	55.56%	34.62%	50.00%	30.77%
Rear quarter = SLOWESTQ	42.31%	61.54%	38.46%	65.38%	44.44%	65.38%	50.00%	69.23%
Front SLOWESTQ$_{TIME}$ same as rear	23.08%	3.85%	15.38%	3.85%	14.81%	3.85%	15.38%	3.85%
SLOWESTQ poor MPC vacuum phase contrast	26.92%	15.38%	26.92%	15.38%	29.63%	15.38%	23.08%	15.38%
TEAT$_{DIAMETER}$ of SLOWESTQ (mm)	29.40	31.46	30.00	31.67	29.80	31.67	28.54	31.80
PARITY	2.27	3.27	2.00	3.35	2.15	3.38	2.15	3.27
DIM	33.04	32.08	30.88	33.54	31.89	32.92	31.85	34.08
YIELD (kg)	15.66	17.71	15.05	18.04	14.83	18.12	14.77	18.26
MACHINEON$_{TIME}$ (s)	419.23	525.46	439.00	539.92	420.11	535.96	421.31	547.81
VD$_{OM}$ (s)	57.65	167.54	64.96	154.50	72.59	156.58	70.88	131.92
LOWFLOW$_{TIME}$ (s)	88.81	176.42	103.84	173.19	103.26	170.35	107.73	145.40
HIGHFLOW$_{TIME}$ (s)	301.65	317.85	283.81	336.96	285.63	336.69	282.88	353.08
DEAD$_{TIME}$ (s)	4.62	4.23	4.62	4.81	3.70	4.62	3.46	4.42
SMT$_{TOT}$ (kPa)	35.09	35.96	35.58	35.99	35.51	35.95	35.62	36.01
SMT$_{PFP}$ (kPa)	34.11	33.76	34.52	34.13	34.28	34.05	34.42	34.50
SMT$_{OM}$ (kPa)	41.19	40.17	41.59	40.18	41.48	40.09	41.54	40.45
MPC$_{TOT}$ (kPa)	31.07	25.85	31.90	25.01	31.13	24.95	31.78	24.97
MPC$_{PFP}$ (kPa)	30.26	22.83	31.18	22.20	30.28	22.05	31.00	22.67
MPC$_{OM}$ (kPa)	35.53	31.82	35.83	31.78	35.29	31.61	35.17	31.49
ADOD_VD$_{OM}$Q23_ACR$_{OM}$	54.77	195.65	32.71	162.02	26.09	138.71	17.19	117.29

Supplementary Table S5: Teat length measurements (mm) by parity group (1, 2, 3, ≥4) for front quarters (Q12), rear quarters (Q34), and all four quarters (Q1234)

**Parity**	**Front quarters (Q12)**			**Rear quarters (Q34)**			**All four quarters (Q1234)**		
	Mean	Std Dev	Range	Mean	Std Dev	Range	Mean	Std Dev	Range
1	46.10^{b}	7.11	30.00	37.40^{b}	6.79	30.00	41.75^{b}	5.82	26.25
2	49.31^{a}	9.42	32.50	39.31^{a}	7.35	30.00	44.31^{a}	7.55	30.00
3	51.81^{a}	8.22	37.50	39.44^{a}	7.30	25.00	45.63^{a}	7.20	30.00
4+	53.26^{a}	12.80	42.50	42.93^{a}	9.25	35.00	48.10^{a}	10.39	37.50

$^{a\text{-}c}$ Mean teat length values in the same column with different superscripts differ ($p < 0.05$) for the interaction between quarter combination and parity group

Supplementary Table S6: Teat diameter measurements (mm) by parity group (1, 2, 3, ≥4) for front quarters (Q12), rear quarters (Q34), and all four quarters (Q1234)

**Parity**	**Front quarters (Q12)**			**Rear quarters (Q34)**			**All four quarters (Q1234)**		
	Mean	Std Dev	Range	Mean	Std Dev	Range	Mean	Std Dev	Range
1	28.30^{b}	3.29	15.00	27.30^{c}	3.30	12.50	27.80^{c}	3.07	13.75
2	30.52^{a}	3.50	12.50	30.00^{b}	3.78	15.00	30.26^{b}	3.31	13.75
3	32.50^{a}	5.00	15.00	31.67^{ab}	4.37	15.00	32.08^{ab}	4.60	15.00
4+	32.50^{a}	3.02	12.50	32.72^{a}	3.84	15.00	32.61^{a}	3.26	13.75

$^{a\text{-}c}$ Mean teat diameter values in the same column with different superscripts differ ($p < 0.05$) for the interaction between quarter combination and parity group
